# Polymorphism, negative thermal expansion and electrical properties of SrNb_2_O_6_

**DOI:** 10.1039/d6tc01467d

**Published:** 2026-09-16

**Authors:** Sadi Ege Parim, Thomas E. Hooper, Lewis R. Owen, Derek C. Sinclair

**Affiliations:** a School of Chemical, Materials and Biological Engineering, Sir Robert Hadfield Building, University of Sheffield Mappin Street Sheffield S1 3JD UK separim1@sheffield.ac.uk d.c.sinclair@sheffield.ac.uk

## Abstract

A combination of variable temperature X-ray diffraction, thermal analysis, and electrical measurements by fixed frequency dielectric spectroscopy, impedance spectroscopy, and electro-motive force measurements are used to establish the structure–electrical properties of SrNb_2_O_6_ ceramics prepared by the conventional solid state reaction technique. A polymorphic phase transition from a monoclinic, m, (space group *P*2_1_/*c*) to an orthorhombic, o, (space group, *Pmcn* that is isomorphic with *Pnma*, CaTa_2_O_6_) cell occurs at ∼420 °C, accompanied by a negative thermal expansion effect (NTE) along the *a*-axis. The NTE in the monoclinic phase is driven primarily by a buckling of Nb_2_O_10_ dimers linked by corners sharing anions along the *b*-axis combined with a switch in their canting arrangement along the *c*-axis across the m- to o-transition. SrNb_2_O_6_ ceramics show no evidence of ferroelectricity; however, they display dielectric behaviour below ∼300 °C with m-SrNb_2_O_6_ exhibiting a room temperature permittivity of ∼30 that approximately doubles on transformation to o-SrNb_2_O_6_. There is significant leakage conductivity associated with adsorption of moisture near room temperature and mixed oxide-ion and p-type electronic conductivity at >300 °C. Both of these space charge related mechanisms are possible origins of the anomalous behaviour in the dielectric spectroscopy measurements that give rise to the apparent ferroelectric behaviour and mask the intrinsic electrical properties of SrNb_2_O_6_.

## Abstract 2

The crystal structure–electrical properties of SrNb_2_O_6_ are established. A polymorphic phase transition from a monoclinic (*P*2_1_/*c*) to an orthorhombic (*Pmcn*) cell occurs at ∼420 °C, accompanied by a Negative Thermal Expansion effect along the *a*-axis.

## Graphical abstract

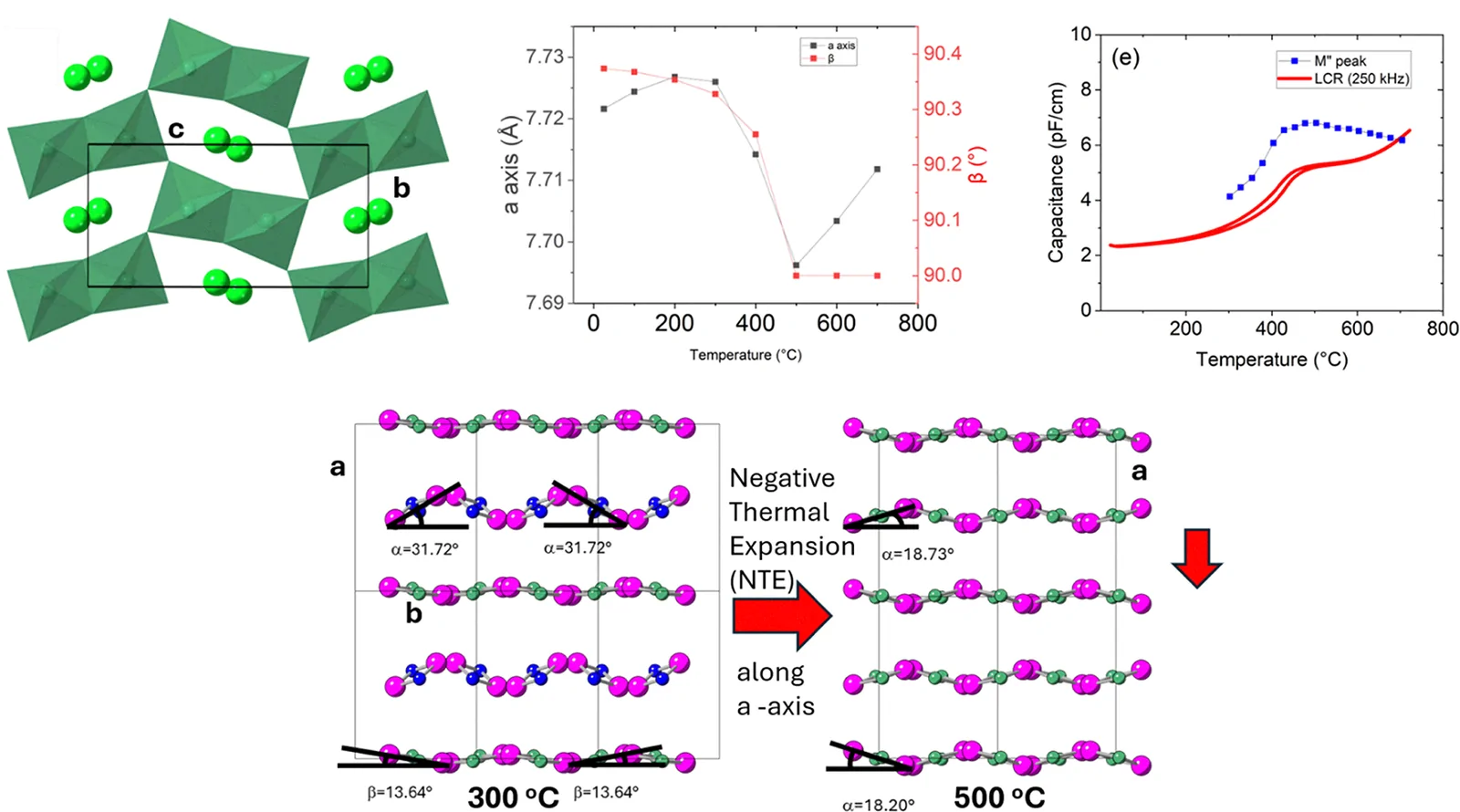

## Introduction

There are many compounds that form with the general formula AB_2_O_6_ where A and B are based on main group and transition metal cations.^1^ Although a broad range of crystal structures exist for such compounds many can be rationalised on the basis of a near hexagonally close packed (hcp) array of oxide ions with 50% of the octahedral holes occupied by the A and B cations. Many different distributions of the A and B cations are possible and depending on the ion requirements several structural variants are possible including columbite, trirutile and rosiaite. The group–subgroup relationships based on lowering of the symmetry and the factors that influence the distribution of the cations in each of these variants has been reviewed by Beck.^2,3^ When A-site cations are too large to occupy the octahedral sites, the structural possibilities become more bespoke. The alkaline earth niobates, AENb_2_O_6_ where AE = Mg, Ca, Sr, and Ba are a good example of this trend. The smaller Mg^2+^ and Ca^2+^ ions form in the Columbite structure (space group, *Pbcn*) where both the AE and Nb^5+^ ions reside in octahedral sites^2,3^ whereas the larger Sr^2+^ and Ba^2+^ ions form different structures based on co-ordination numbers of 8 for Sr (monoclinic (m)-SrNb_2_O_6_ (*P*2_1_/*c*)^4^ or orthorhombic (o)-SrNb_2_O_6_ (*Pnma*)^5^) and 8, 10, or 12 for the Ba analogues: o-BaNb_2_O_6_-I (*Pmma*),^6^ m-BaNb_2_O_6_ (*P*2_1_/*c*)^7^ and o-BaNb_2_O_6_-II (*C*222_1_),^8^ respectively.

There have been several studies on the synthesis, structure and properties of SrNb_2_O_6_ since it was reported by Francombe in 1960.^9–14^ This interest primarily stemmed from the ferroelectric (and therefore pyro- and piezo-electric) tetragonal tungsten bronze solid solution (Sr_*x*_Ba_1−*x*_NbO_6_, SBN, *x* = 0.25–0.75) formed between SrNb_2_O_6_ and BaNb_2_O_6_;^9^ however, more recent interest has focussed on its photocatalytic^15–18^ and electro-optic properties.^19–21^

SrNb_2_O_6_ has been prepared in the form of single crystals,^10^ 1-^16^ and 2-D nanostructures^17^*via* hydrothermal methods, and polycrystalline powders by conventional solid-state reaction of SrCO_3_ and Nb_2_O_5_.^9,11–14^ Although there is some disparity in the melting point, thermal stability and variety of polymorphic forms of SrNb_2_O_6_ (*e.g.* m-, o- and cubic^12^) depending on synthesis conditions^9–14^ it is generally accepted that at room temperature a monoclinic, pseudo-orthorhombic modification (m-SrNb_2_O_6_) isostructural with orthorhombic CaTa_2_O_6_ (rynersonite, space group *Pnma*)^22^ is formed. In this case the structure of m-SrNb_2_O_6_ (with *a* ∼ 7.72, *b* ∼ 5.59, *c* ∼ 10. 99 Å and *β* ∼ 90.37°) can be described as a framework of distorted NbO_6_ octahedra sharing edges and corners with Sr ions in a corrugated (zig-zag) arrangement in channels running in the direction of the *a*-axis, Fig. 1(a). A characteristic feature of the topology is the edge sharing octahedral dimers that are connected to others by sharing all remaining vertices. The vertex sharing of the dimers within the {100} planes and out of plane in the [911] direction to show the Nb(1) and Nb(2) ions is illustrated in Fig. 1(b). The columns of dimers are alternatively tilted and create channels for the larger Sr^2+^ ions. A side view along [010] to illustrate the tilting of the vertex sharing octahedra along [001] and the alternating orientation of the SrO_8_ polyhedra is shown in Fig. 1(c). These bi-capped trigonal prismatic SrO_8_ units are connected *via* edge sharing within the channels. The monoclinic distortion has been attributed to the restricted space for the Sr^2+^ ions in the tunnels. A recent Rietveld refinement study of laboratory X-ray diffraction (XRD) data over the range 25 to 700 °C showed no evidence of any polymorphic transition in m-SrNb_2_O_6_ prepared by a solid-state reaction method, suggesting if there is a m- to o-polymorphic transition it must be above 700 °C.^23^

**Fig. 1:**
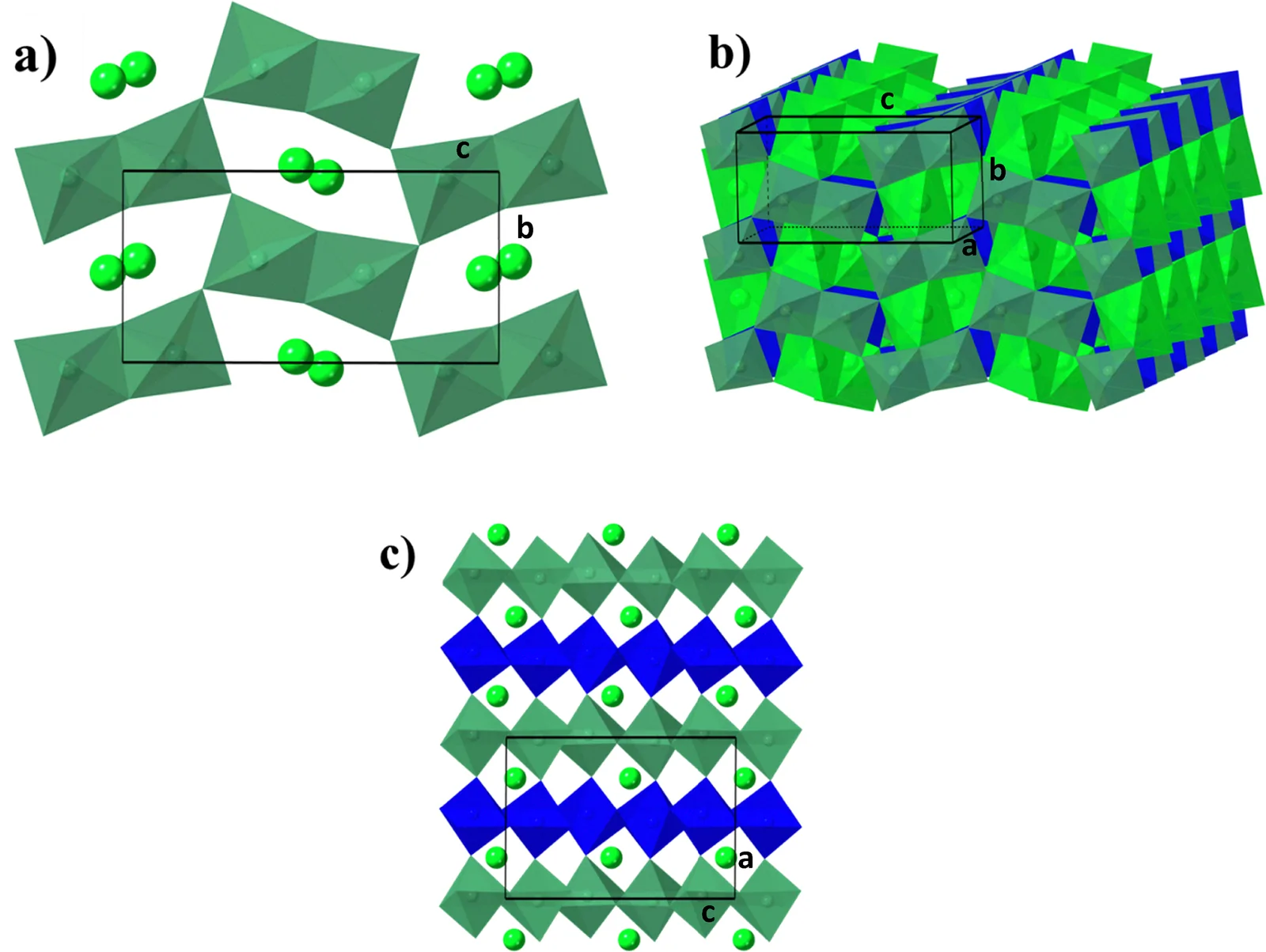
The room temperature monoclinic structure of SrNb_2_O_6_ in a view along (a) [100], (b) offset in [100] to show Nb(1)O_6_ and Nb(2)O_6_ octahedra, and (c) [010]. In (a) Sr atoms are shown in green spheres and NbO_6_ octahedra in olive. In (b) SrO_8_ polyhedra are shown in green and Nb(1)O_6_ and Nb(2)O_6_ octahedra are in olive and blue, respectively. In (c), Sr atoms are shown in green spheres and Nb(1)O_6_ and Nb(2)O_6_ octahedra are in olive and blue, respectively.

There are various conflicting reports on the electrical properties of SrNb_2_O_6_ ceramics.^23–25^ Fixed frequency dielectric spectroscopy (DS) studies have shown a single peak in permittivity and tan *δ* near room temperature^23,24^ or ∼300 °C.^25^ The permittivity and tan *δ* maxima at these reported peaks are very frequency dependent and decrease substantially with increasing frequency. For example, in the study by Pastor *et al.*^24^ where the permittivity peak maximum occurs at ∼110 °C, permittivity and tan *δ* decrease from ∼570 and 1.5 at 1 kHz to ∼325 and 0.2 at 100 kHz. Some of these reports^23–25^ attribute the peak in permittivity to a ferroelectric to paraelectric transition but this is inconsistent with structural analysis where the room temperature XRD data are fitted to *P*2_1_/*c* which is a centrosymmetric space group. On this basis, ferroelectricity and high permittivity are not expected for SrNb_2_O_6_. The origin(s) of these various dielectric anomalies from the reported DS data are therefore unresolved and the structure–dielectric properties of m-SrNb_2_O_6_ remain to be established.

Here we use a combination of variable temperature XRD, scanning electron microscopy (SEM), differential scanning calorimetry (DSC), DS and impedance spectroscopy (IS) to resolve many of the reported discrepancies in the literature to establish the structure–electrical (dielectric and transport) properties of SrNb_2_O_6_ ceramics prepared from a conventional solid state reaction method. We present evidence for a m- to o-polymorphic phase transition at ∼420 °C that is accompanied by a negative thermal expansion effect along the *a***-**axis of the cell and a doubling of the permittivity from ∼28 at RT for m-SrNb_2_O_6_ to ∼60 for o-SrNb_2_O_6_ at ∼600 °C. The ceramics exhibit modest levels (∼10^−8^ to 10^−4^ Scm^−1^) of mixed electronic (p-type) and ionic (oxide ion) conductivity between ∼300 and 800 °C. This leakage conductivity associated with the electrical transport properties at >300 °C dominates the lower frequency DS data in the vicinity of the m- to o-polymorphic phase transition and IS is required to establish the intrinsic (bulk) permittivity-temperature characteristics of the SrNb_2_O_6_ polymorphs.

## Experimental

SrNb_2_O_6_ was prepared by the conventional mixed oxide route using starting reagents of SrCO_3_ (99.9%, Sigma Aldrich) and Nb_2_O_5_ (99.5%, H.C. Starck). The reagents were dried at 180 and 900 °C prior to batching. Powders were mixed with a 1 : 1 molar ratio and ball-milled for approximately 24 hours with yttria-stabilised zirconia milling media using isopropanol as a solvent. The mixture was dried in a Kenwood mixer and sieved with a 300 µm sieve. The powders were then placed in alumina crucibles and calcined at 1100 °C for 8 hours using a 5 °C min^−1^ heating rate in air. The calcined powders were then ball-milled and dried for a second time with the same process and the sieved powders pressed with a uniaxial cold press using a 10 mm diameter steel die at 25 MPa. The samples were then cold isostatic pressed at ∼200 MPa (30 kpsi) for 2 min. The green body pellets were sintered in a box furnace in air at 1350 °C for 8 h with 5 °C min^−1^ heating rate.

The structural and phase analysis of crushed powders from the sintered ceramics was carried out using XRD. High (variable) temperature XRD (VTXRD) from 25 °C (RT) to 700 °C was performed using a high precision PanalyticalXPERT^3^ diffractometer and Cu Kα radiation with *λ* = 1.5406 Å. Analysis was operated with 45 kV, 40 mA with a step size of 0.26° and count time of 90 s per step over a 2*θ* range from 10 to 90 degrees. Diffraction patterns were collected on heating with 30 °C min^−1^ heating rate in the temperature range of 100 to 700 °C at 100 °C increments with dwell times of 20 min at each temperature to ensure thermal equilibration prior to data collection. Rietveld refinements were performed by using GSAS-II software^26^ and parameters of the refinements consist of lattice parameters, atomic coordinates, site occupancies, thermal parameters (cations only as anion values were fixed) and crystallite size. For both XRD and VTXRD samples were prepared by crushing sintered pellets with a pellet crusher and grinding the powder using a pestle and mortar. To remove residual stress, the powders were annealed at 600 °C with a 5 °C min^−1^ heating rate, 1 h dwell time and 0.8 °C min^−1^ cooling rate prior to measurements.

Scanning electron microscopy (SEM) was used to establish the microstructure of the sintered ceramics. As-sintered pellets were ground sequentially with P400, P800, P1200 and P2500 grade silicon carbide papers. The thinned pellets were then polished sequentially with 6, 3 and 1 µm diamond solution with silk paper. The samples were then thermally etched at 1150 °C for 15 minutes. Etched samples were mounted on 10 mm steel stubs with silver dag and carbon coated. Analysis was performed using an FEI Inspect F50 microscope with secondary electron, backscattered electron and energy dispersive X-ray analysis (EDX) methods being used. 20 kV high voltage was applied with a ∼10 mm working distance, spot size of 3 and 5000× magnification used for all imaging processes.

Differential Scanning Calorimetry (DSC) was used to detect any possible phase transitions at high temperatures using a Netzsch DSC 404C Pegasus DSC. 30 mg of crushed pellet were heated up to 800 °C and cooled down to 20 °C with 10 °C min^−1^ rate for 3 cycles in argon atmosphere in an alumina crucible and an empty alumina crucible used as a reference.

Dielectric spectroscopy (DS) analysis was performed using an Agilent Precision LCR Meter to acquire relative permittivity (*ε*_r_) and dielectric loss (tan *δ*) as a function of temperature. Electroded pellets were inserted in an impedance rig within a tube furnace and heated and cooled with a scan rate of 1 °C min^−1^ from room temperature to 700 °C and measured with a frequency range of 1 kHz to 1 MHz in air. Prior to electrical measurements, organo-gold paste was applied to the major faces of the pellets and heated to 850 °C with a 2 h dwell time. All measurements were corrected for sample geometry based on the thickness of the sample and the electrode area.

Impedance spectroscopy (IS) was used to determine conduction type, conduction properties and activation energy calculations for bulk conduction. A similar furnace setup as LCR was used with gold electrode pellets and measurements taken 30 mHz to 2 MHz frequency range between the temperature range of 100–750 °C with 25 °C increments manually and both the Agilent and Solarton® instruments were used for measuring *M*″ spectra in a frequency range of 30 mHz to 2 MHz in air atmosphere. A nitrogen atmosphere (oxygen-fee N_2_, OFN, with an O_2_ level of 5 ppm) was used for p–n type determination. Measured impedance data were corrected with the geometrical factor and analysed with Z-view commercial software (Scribner Associates, Inc., Charlottesville, VA, version 4.0).

Electromotive force (EMF) measurements were performed to determine the oxide ion transport number (*t*_o_) in SrNb_2_O_6_ ceramics. Measurements were performed using a ProboStat system (NorECs Norwegian Electro Ceramics AS, Oslo, Norway). The as-sintered ceramic was glued onto a YSZ tube by using a commercial glass frit. A difference in oxygen partial pressure (pO_2_) was created by flowing N_2_ gas into the YSZ tube with an air atmosphere outside the tube. The difference in pO_2_ was determined by voltage readings between the inner and outer electrodes of the YSZ tube. Voltage measurements were recorded using a Rapid© commercial digital multimeter.

## Results

Room temperature XRD data of crushed powder from sintered ceramics of SrNb_2_O_6_ fully indexed on a monoclinic cell (space group with *P*2_1_/*c*) with *a* = 7.7217(2), *b* = 5.5937(1), *c* = 10.9829(3) Å and *β* = 90.374(2)°, Fig. 2(a) and SI Tables S1(a) and S2. DSC of crushed powder from the sintered ceramics showed the presence of an endotherm on heating at ∼420 °C and an exotherm at ∼410 °C on cooling, Fig. 2(b), indicating the presence of a phase transition in the material. XRD data collected on a sample at 700 °C fully indexed on an orthorhombic cell (space group *Pmcn*) with *a* = 7.7118(1), *b* = 5.6744 (1) and *c* = 11.0293(2) Å, Fig. 2(c) and SI Tables S1(b) and S2. VTXRD data between RT and 700 °C are shown in Fig. 3(a) and on an expanded scale in Fig. 3(b). The data sets between RT and 400 °C fully indexed on the monoclinic cell with *β* decreasing to 90.225(2)°, whereas the data sets from 500 to 700 °C fully indexed on an orthorhombic cell (space group *Pmcn*). The decreasing peak splitting associated with the (013) and (211) reflections for the o-polymorph at 500 °C compared to that obtained for the m-polymorph below the transition is shown clearly in Fig. 3(b). Details of the Rietveld refinements for these data sets are provided in SI Fig. 1 and SI Table S2.

**Fig. 2:**
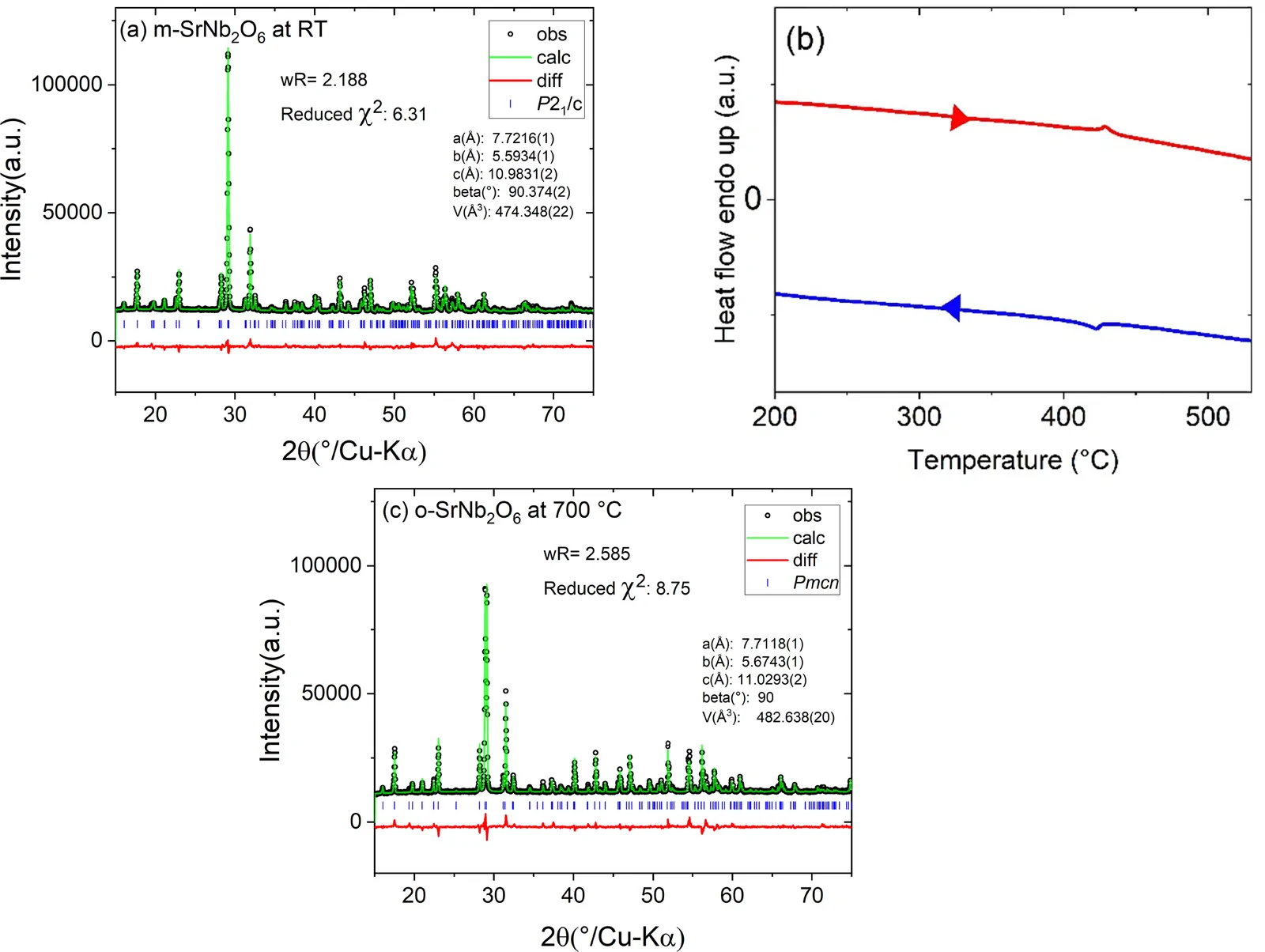
XRD patterns of crushed powder from sintered SrNb_2_O_6_ ceramics at (a) RT and (c) 700 °C. (b) DSC *versus* heating and cooling for the crushed SrNb_2_O_6_ powder.

**Fig. 3:**
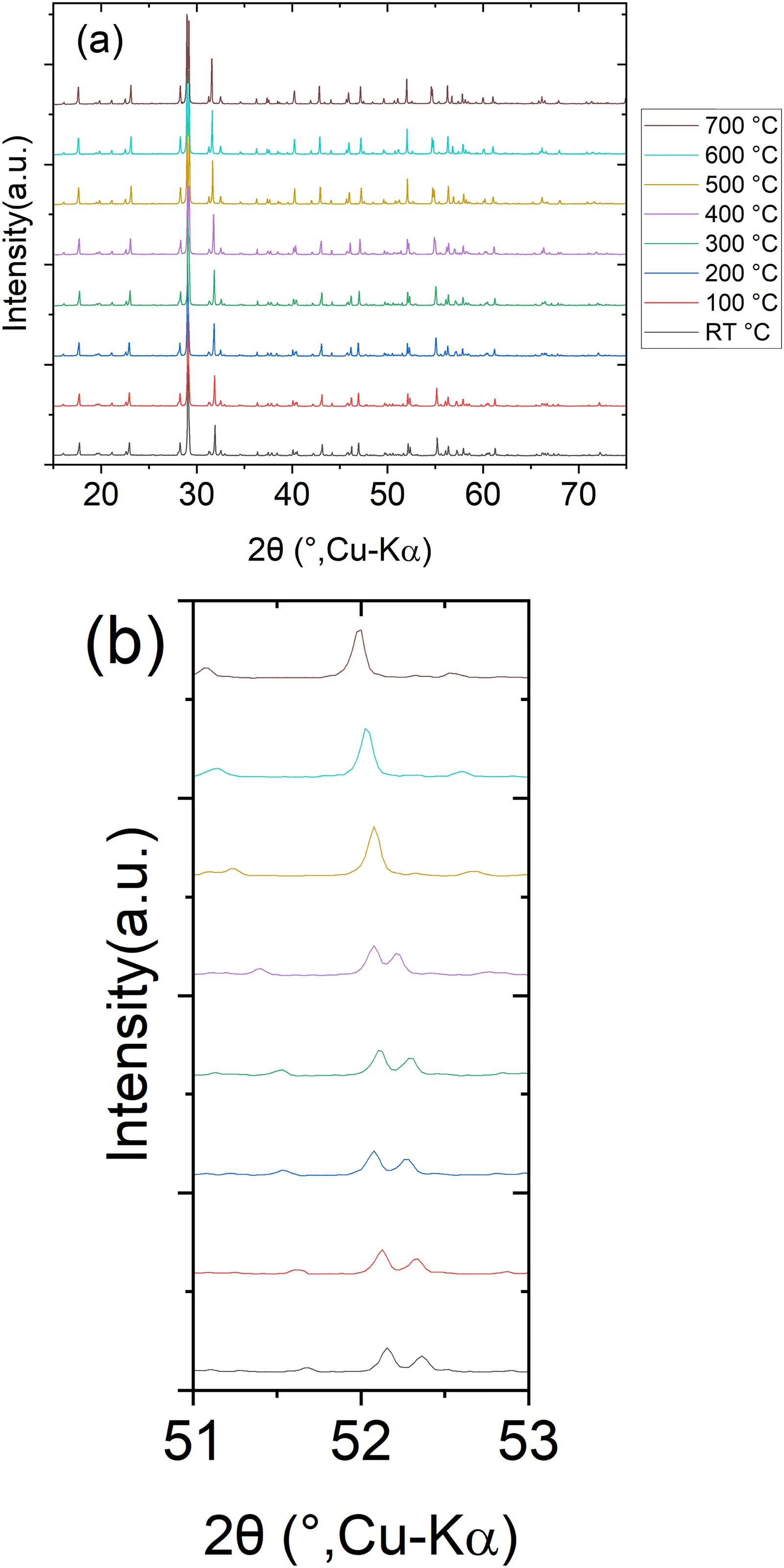
(a) VTXRD patterns of crushed powder from sintered SrNb_2_O_6_ ceramics between RT and 700 °C. (b) Expanded scale of the VTXRD data in the range 51 to 53° 2*θ*.

The variation in the lattice parameters, *β* and cell volume are shown in Fig. 4. The trend lines are to guide the eye for the variations in the monoclinic and orthorhombic cells as opposed to indicate where the phase transition is occurring which is between the VTXRD data sets collected at 400 and 500 °C. The unit cell *a*-axis initially expands on heating to ∼200 °C but then exhibits a range of negative thermal expansion (NTE) before expanding again at ∼500 °C, Fig. 4(a). The *b* and *c* axes both show expansion on heating; the variation of *b* is nearly linear whereas *c* increases rapidly up to ∼500 °C before levelling-off, Fig. 4(b) and (c), respectively. *β* decreases from 90.374(2)° at RT to 90° at 500 °C, Fig. 4(d). There is a general increase in volume of the cell with temperature, despite the slight negative thermal expansion in the *a*-axis noted previously; however, there is a change in the slope of the cell volume *versus* temperature between 400 and 500 °C, Fig. 4(e). The variation in the average Sr–O and Nb–O bond lengths with temperature show an expansion with temperature for the m- and o-polymorphs; however, their temperature dependence is different, Fig. 5. There is a notable expansion in the average Sr–O bond within the SrO_8_ polyhedra from RT to 700 °C but with some levelling-off between 300 and 500 °C across the phase transition, Fig. 5(a). In contrast, there is modest expansion of average Nb–O bond length within the dimers in the measured temperature range with only Nb(1)–O showing a clear increase below the phase transition temperature, Fig. 5(b). Details of selected bond lengths and angles from the Rietveld refinement analysis of the data for data sets at RT, 300, 500 and 700 °C are provided in SI Table S3. Attempts to stabilise o-SrNb_2_O_6_ at RT by rapid quenching of powder from 1000 °C into liquid nitrogen using a quench furnace were unsuccessful; transformation to m-SrNb_2_O_6_ always occurs, see SI Fig. S3.

**Fig. 4:**
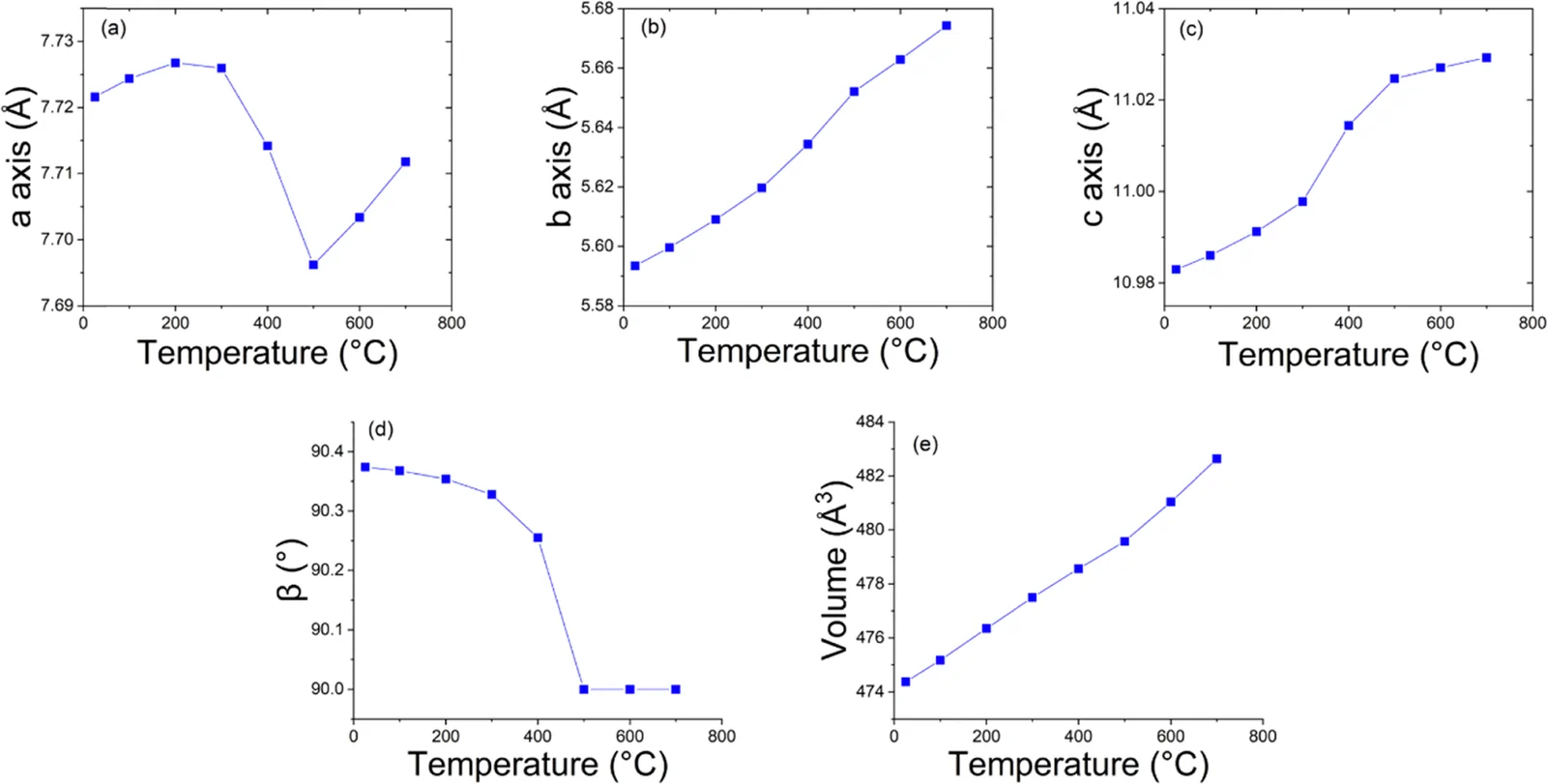
Variation of lattice parameters (a–c), *β* (d) and cell volume (e) *versus* temperature for SrNb_2_O_6_.

**Fig. 5:**
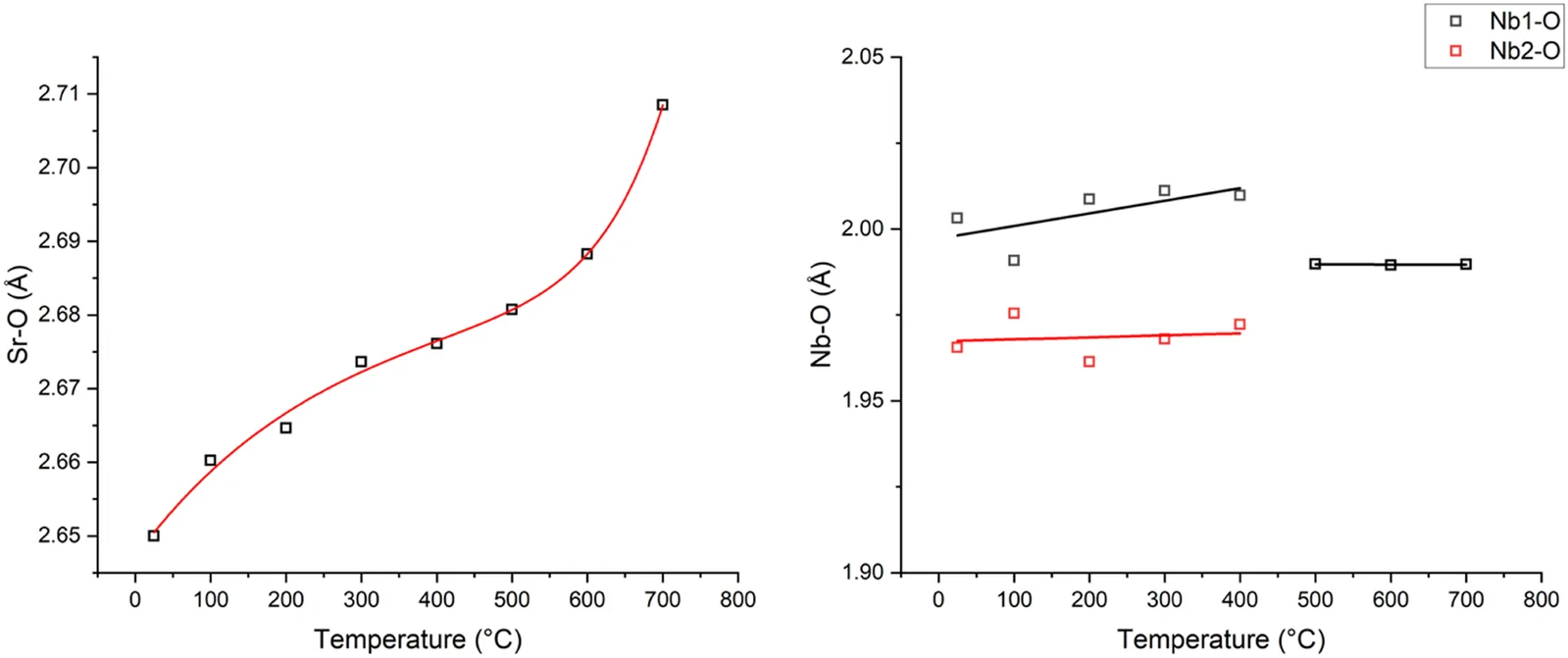
Variation of average Sr–O (a) and Nb–O (b) bond lengths *versus* temperature for SrNb_2_O_6_.

Sintered ceramics were ∼92% of the theoretical density calculated from refined XRD data lattice parameters, with the grain size typically in the range of ∼1–5 microns, SI Fig. 2. DS showed the ceramics to have RT permittivity and tan *δ* of ∼28 and 0.003, respectively. The permittivity and tan *δ* start to increase above 200 °C and become frequency dependent, Fig. 6(a) and (b). There is an apparent peak in permittivity at ∼500 and 675 °C at 1 and 10 kHz, respectively, Fig. 6(a). Closer inspection of the 100 kHz to 1 MHz data show evidence of a permittivity plateau at ∼500 °C, Fig. 6(c) before starting to increase again at higher temperatures. The DS behaviour is reversible on heating and cooling with only a low level of hysteresis between ∼200 and 500 °C, Fig. 6(c) and (d). To investigate the possibility of moisture uptake by the ceramics being responsible for the upturn in tan *δ* near room temperature and at lower frequencies, Fig. 6(a) a sample at RT had the circumference coated with Vaseline.^27^ The permittivity showed a modest increase, Fig. 6(e) whereas there was a substantial reduction in tan *δ* near RT, Fig. 6(f). The ability of Vaseline to absorb moisture and eliminate short circuit pathways between the electrodes *via* the outer surfaces of the ceramics highlights that humidity may strongly influence the DS measured dielectric properties of SrNb_2_O_6_ near RT.

**Fig. 6:**
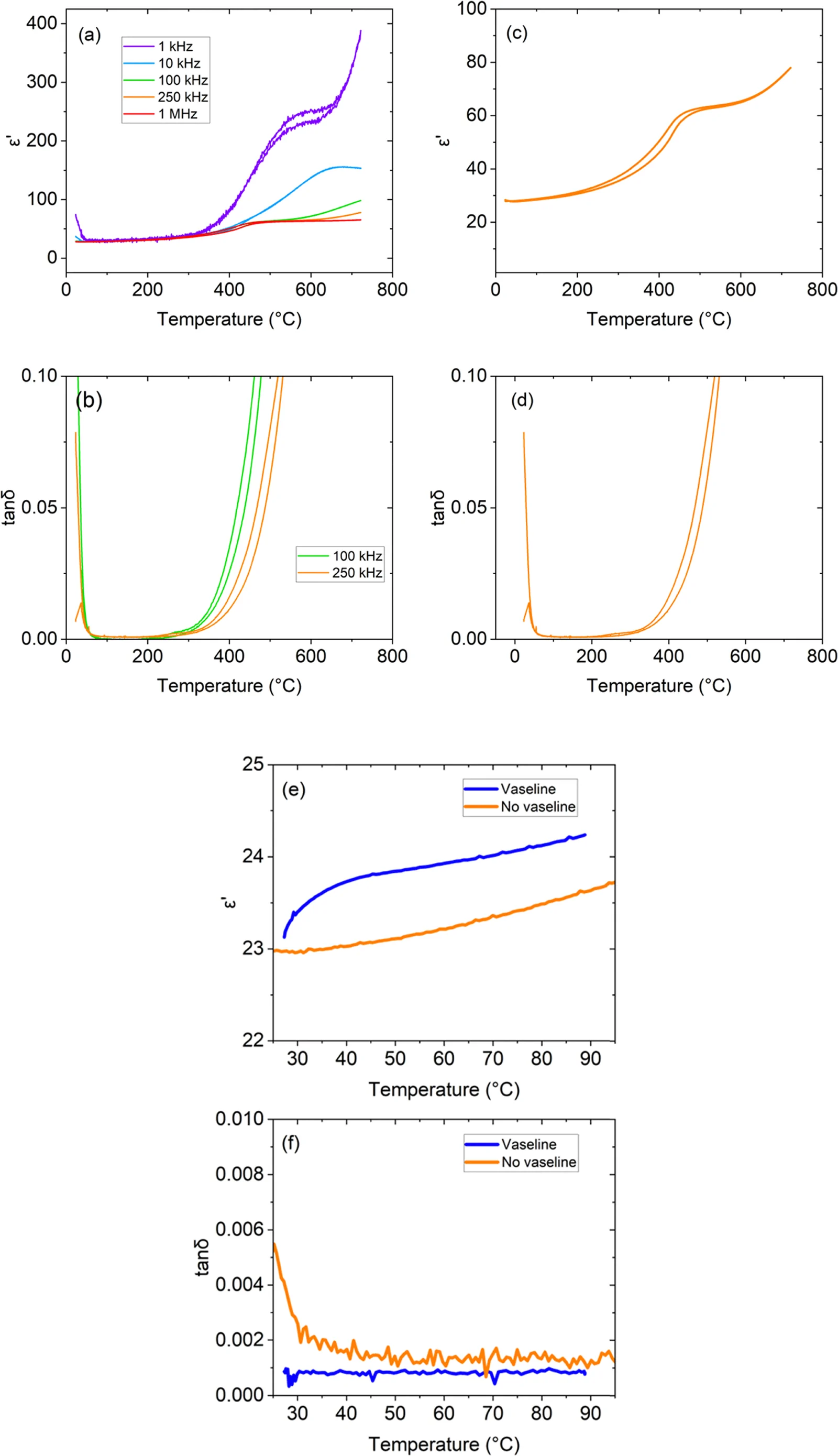
DS data for SrNb_2_O_6_ ceramics. Permittivity and tan *δ versus* temperature as a function of frequency (a) and (b), respectively, and as a function of heating and cooling at 250 kHz, (c) and (d), respectively. DS data *versus* temperature near RT for samples with and without Vaseline being applied to the outer surfaces of the ceramics between the electrodes prior to measurements, (e) and (f).

IS was performed between ∼300 and 700 °C to establish the high temperature electrical properties of the sintered ceramics. Complex impedance plane, Z*, plots for a sample measured at ∼640 °C in air consisted of a broad, asymmetric arc and a low frequency tail, Fig. 7(a). Lowering the oxygen partial pressure (pO_2_) from laboratory air to flowing N_2_ gas resulted in an increase of overall resistivity from ∼160 to 230 kΩcm indicating the ceramics exhibit p-type conduction, Fig. 7(a). The capacitance associated with the Z* arc was ∼7 pF cm^−1^ which is consistent with a dominant bulk (grain) response with no significant influence of any grain boundary contribution which would be expected to have a much higher associated capacitance in the range of nF cm^−1^.^28^ The corresponding combined −*Z*″, *M*″ spectra show evidence of single, broad peaks with *f*_max_ values that differ by less than half an order of magnitude, based on a log_10_(*f*) scale, Fig. 7(b). Changing the pO_2_ from air to N_2_ has minimal effect on the *f*_max_ values, however the magnitude of the −*Z*″ peak increases significantly which is consistent with the Z* plots. Based on this information, the IS data were modelled (to a first approximation) on a single, parallel RC element associated with a grain (bulk) response connected in series with a Warburg-type element for the low frequency tail in Z* plots associated with an electrode response.

**Fig. 7:**
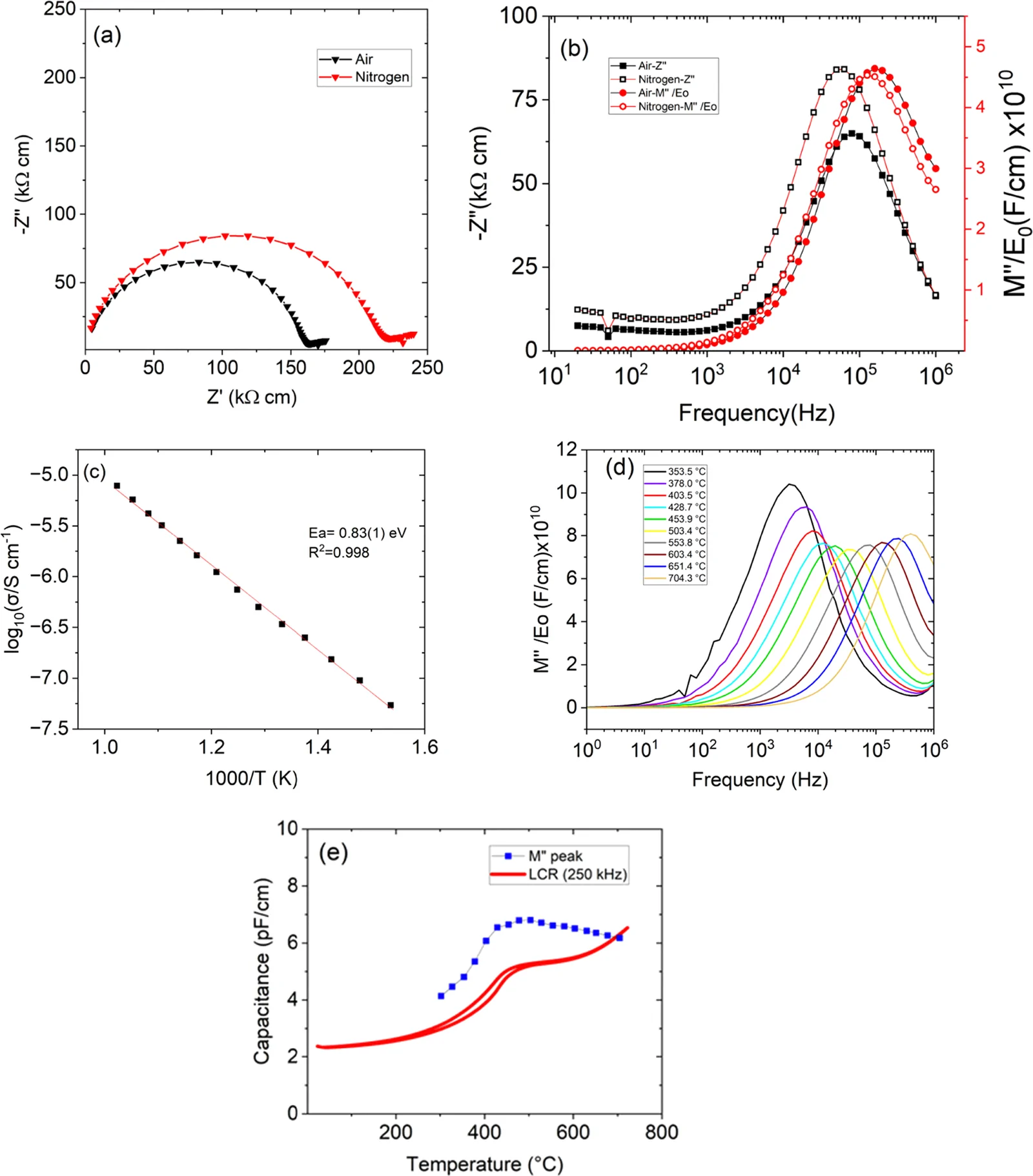
IS data and extracted conductivity and capacitance values for SrNb_2_O_6_ ceramics. Z* (a) and combined −*Z*″, *M*″ spectra (b) in air (open symbols) and flowing N_2_ gas (red) at ∼640 °C. Arrhenius plot of conductivity, *σ*, *versus* reciprocal temperature (c) based on data from Z* plots. *M*″ spectra (d) and extracted *C* values in blue compared with DS at 250 kHz (e) for the temperature range ∼400 to 700 °C.

The resistivity of the SrNb_2_O_6_ ceramics measured in laboratory air was extracted from the intercept of the arc on the *Z*′ axis in the Z* plots, Fig. 7(a). The conductivity, *σ*, was calculated from the inverse of the resistivity values and plotted in Arrhenius-format as shown in Fig. 7(c). Linear behaviour was obtained over the measured temperature range with an associated activation energy for conduction of ∼0.83(1) eV. Given the sensitivity of the electric modulus formalism to highlight elements of low capacitance and therefore bulk responses,^28^ the relationship 
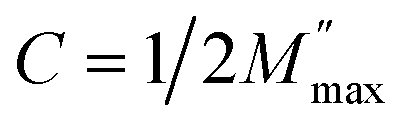
 was used to extract *C* values for the ceramics across the measured temperature range. *M*″ spectra at various temperatures are shown in Fig. 7(d) and the extracted *C* values in Fig. 7(e) are compared to that obtained from DS. The minimum in *M*″ peak height at ∼500 °C (and therefore the maximum in C) are in reasonable agreement with the changes in the VTXRD, DSC and DS data between ∼400 and 500 °C. Electromotive force measurements performed between 600 and 800 °C established *t*_o_ to be ∼0.26 and 0.31, see SI Fig. S4. Combined with the pO_2_ IS data these results indicate mixed electronic–ionic conduction to be present at elevated temperatures in SrNb_2_O_6_ ceramics.

## Discussion

The RT XRD data in Fig. 2(a) confirm previous reports of a monoclinic cell (space group *P*2_1_/*c*) for SrNb_2_O_6_ prepared by solid state reaction. Bond valence sums^29^ based on the Sr–O and Nb–O lengths given in SI Table S1(a) yield values of 1.99 vu for Sr and 4.89 and 5.52 vu for Nb(1) and Nb(2), respectively. This indicates acceptable bonding environments for Sr and Nb(1) ions but significant over-bonding for Nb(2) ions. The flexibility in the structure primarily resides with the corner sharing oxygen ions that link the dimers (both in- and out-of-plane, Fig. 1) that facilitates both octahedral tilting and the monoclinic distortion in the unit cell. Combining the VTXRD and DSC data in Fig. 2–4 establish a polymorphic phase transition on heating in SrNb_2_O_6_ to an orthorhombic cell (o-SrNb_2_O_6_) at ∼420 °C where all the Nb ions become equivalent, Fig. 5(b).

Interesting features of the VTXRD data are: (i) the NTE effect observed along the *a*-axis (direction of the edge sharing SrO_8_ polyhedra channels) from ∼200 °C for m-SrNb_2_O_6_ up to the phase transition, Fig. 4(a); (ii) the levelling-off in the average Sr–O bond length within the SrO_8_ polyhedra across the transition from the m- to o-polymorph, Fig. 5(a); and (iii) the increase in the average Nb–O bond length of the NbO_6_ octahedra associated with overbonded Nb(2)O_6_ in m-SrNb_2_O_6_ across the phase transition, Fig. 5(b). Below we try to rationalise these features (in reverse order) to understand the origin(s) of the m- to o-polymorphic transition and the NTE effect.

At this stage it is worth inspecting various cation–cation interactions in the two polymorphs in attempts to understand how movements in the cation sublattice contribute to stabilisation of the polymorphs, Fig. 8. In particular, the various Nb–Nb interatomic distances within the dimers and the interatomic Sr–Sr distances associated with the corrugated arrangement of the Sr ions along the channels. In the m-polymorph at RT, the Nb–Nb distance within the Nb(2)_2_O_10_ dimers is ∼3.16 Å and therefore ∼0.01 Å shorter than that associated with the Nb(1)_2_O_10_ dimers, (∼3.17 Å), Fig. 8(a–i) whereas all Nb–Nb distances within the Nb_2_O_10_ dimers of the o-polymorphic are equivalent, *e.g.* ∼3.2 Å at 700 °C, Fig. 8(a-iv). There is a smooth expansion in these distances at intermediate temperatures, *e.g.* 300 and 500 °C, see Fig. 8(a-ii) and (a-iii), respectively. The Nb(2)–Nb(2) distance in the m-polymorph shows negligible variation (∼3.16 Å, within errors) with temperature up to the phase transition, Fig. 8(b) as does the average Nb(2)–O bond length, Fig. 5(b). The shorter and constrained Nb(2)–Nb(2) distance within the dimers and therefore increased cation–cation repulsion effects is consistent with the shorter average Nb(2)–O bond length and overbonding of Nb(2) cations compared to the Nb(1) ions in the m-polymorph, Fig. 5(b). In contrast there is smooth expansion between the Nb(1) ions within the Nb(1)_2_O_10_ dimers in the m-polymorph and this continues smoothly in the o-polymorph, Fig. 8(b). The changes in the Nb sublattice at the phase transition therefore relieves the cation–cation repulsion effects and compression of the Nb–O bonding associated with the Nb(2) cations in the m-polymorph. Reducing the overbonding within the Nb(2)O_6_ octahedra is presumably a contributing factor for the m- to o-polymorphic phase transition in SrNb_2_O_6_. It is also noteworthy that the concerted movement of cations relieve the short Nb(1)–Sr interatomic distance of ∼3.51 Å in m-SrNb_2_O_6_ at RT as shown by the asterix in Fig. 8a.

**Fig. 8:**
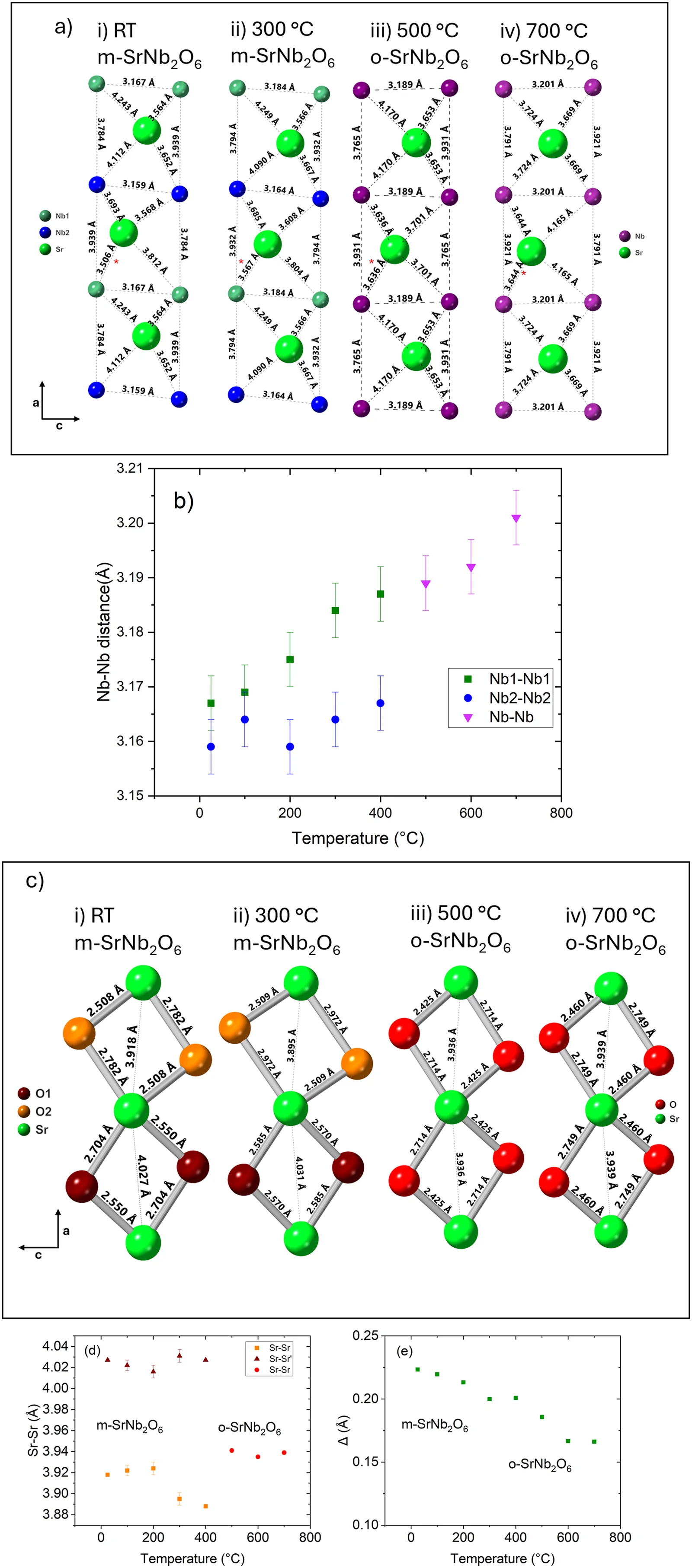
Sr–Nb and Nb–Nb interatomic separations for the Sr and Nb ions in the channels along the *a*-axis (a) in the m-polymorph at RT (i) and 300 °C (ii) and for the o-polymorph at 500 °C (iii) and 700 °C(iv), respectively. Variation in interatomic Nb–Nb distances (b) *versus* temperature for SrNb_2_O_6_. Sr–Sr interatomic separations for the Sr ions in the channels along the *a*-axis (c) in the m-polymorph at RT (i) and 300 °C (ii) and for the o-polymorph at 500 °C (iii) and 700 °C(iv), respectively. Variation in interatomic Sr–Sr distances (d) *versus* temperature for SrNb_2_O_6_. The variation in *Δ versus* temperature (e) for SrNb_2_O_6_.

The connectivity of the Sr ions in the SrO_8_ polyhedra along the channels *via* the edge sharing oxygen ions with selected Sr–Sr interatomic distances and Sr–O bond lengths are shown for the m- and o-polymorphs at RT, 300, 500 and 700 °C in Fig. 8c(i to iv), respectively. In the m-polymorph there are two distinct Sr–Sr interatomic distances, Sr–Sr and Sr–Sr′, Fig. 8c(i and ii), whereas there is only one in the o-polymorph, Fig. 8c(iii and iv). The difference between the Sr–Sr and Sr–Sr′ distances at RT is ∼0.11 Å, *i.e.* ∼3.92 and 4.03 Å, respectively, Fig. 8c(i and ii) and they show anomalous and divergent behaviour from ∼200 °C where the NTE effect starts to occur until above the polymorphic phase transition, Fig. 8c(ii). Initially, Sr–Sr shows a modest expansion from RT to ∼200 °C but then undergoes significant contraction until the phase transition, see Fig. 8(c (ii) and d). In contrast, Sr–Sr′ shows a modest contraction from RT to ∼200 °C followed by a modest expansion, see Fig. 8(c (ii) and d) until the phase transition. Above the phase transition they merge to a single value ∼3.94 Å in the o-polymorph, Fig. 8(c (iii) and (iv), and d).

Although there is expansion in the average Sr–O bond length in the SrO_8_ polyhedra for both polymorphs, there is a significant decrease around the phase transition, Fig. 5(a). If the Sr ions were vertically aligned in the channels the Sr–Sr interatomic distance would be *a*/2. We therefore define *Δ*_m_= [{Sr–Sr + Sr–Sr′} − *a*] and *Δ*_o_ = [2{Sr–Sr} − *a*] as a measure of the extent of the corrugated arrangement of the Sr ions in these channels within the polymorphs and this is shown to decrease with increasing temperature, Fig. 8(e). There is clearly more translational movement of the Sr ions in m-SrNb_2_O_6_ within the SrO_8_ polyhedra and this gives rise to the larger *Δ* associated with the corrugated arrangement of the Sr ions and facilitates the attainment of a suitable bonding environment for the Sr ions within the SrO_8_ polyhedral network as noted by the bond valence sum value of 1.99 for Sr in m-SrNb_2_O_6_, SI Table S1(c).

The NTE effect in the *a*-axis from ∼200 °C to above the phase transition is similar in nature to that observed along the polar *c*-axis for the ferro- to *para*-electric phase transition in the TTB materials, Sr_2_NaNb_5_O_15_ and Ba_2_NaNb_5_O_15_ which has been attributed to changes in corrugation of the A-site cations and polar displacements of the Nb ions along the *c*-axis.^30,31^ SrNb_2_O_6_ is not ferroelectric so there are no significant polar displacements of the Nb ions and therefore the NTE effect must have a different origin. Here we discuss the role of the Nb_2_O_10_ dimers to explain the *a*-axis NTE effect. In particular, a reduction in their buckling along the *b*-axis combined with a switch in their canting arrangement along the *c*-axis across the m- to o-transition.

In m-SrNb_2_O_6_ at 300 °C, there is significant buckling of the alternating layers of Nb(1)_2_O_10_ and Nb(2)_2_O_10_ dimers in opposite directions with respect to the *b*-axis, as shown by the AB plane in Fig. 9(a). This is particularly severe for Nb(2)_2_O_10_ with angles of ∼31.72° compared to ∼13.64° for Nb(1)_2_O_10_. All dimers are canted with respect to the *c*-axis in the same direction (as defined by the angle between the 2 Nb ions in the dimers with the *c*-axis in the AC plane and indicated as +(counter clockwise rotation, ccw) with angles of ∼9.13 and 7.12° for Nb(1)_2_O_10_ and Nb(2)_2_O_10_, respectively, Fig. 9(b). In o-SrNb_2_O_6_ at 500 °C where all Nb ions are equivalent, the alternating layers of dimers in the AB planes are still in opposite directions with respect to the *b*-axis but the overall buckling is significantly reduced with equivalent angles of ∼18.20°, Fig. 9(c). In addition, there is switch in the orientation of the canting of the dimers in the AC planes where it is now + (ccw) and − (clockwise rotation, cw) between successive layers with smaller and equal angles of ∼5.94°, Fig. 9(d). The Nb and O movements within the AB and AC planes that create these changes in angles associated with the dimers result in the primary contraction of the *a*-axis as SrNb_2_O_6_ undergoes the m- to o-phase transition. It should be noted that these canting mechanisms of the Nb_2_O_10_ dimers also influence the corrugation of the Sr ions within the SrO_8_ units given all polyhedral units have some common, shared anions. It is clear that higher quality diffraction data *via* synchrotron XRD and powder Neutron Diffraction data (for accurate positions of the oxygen ions) combined with symmetry mode analysis would provide greater insight to the origin(s) of the polymorphic phase transition and the NTE effect in SrNb_2_O_6_.

**Fig. 9:**
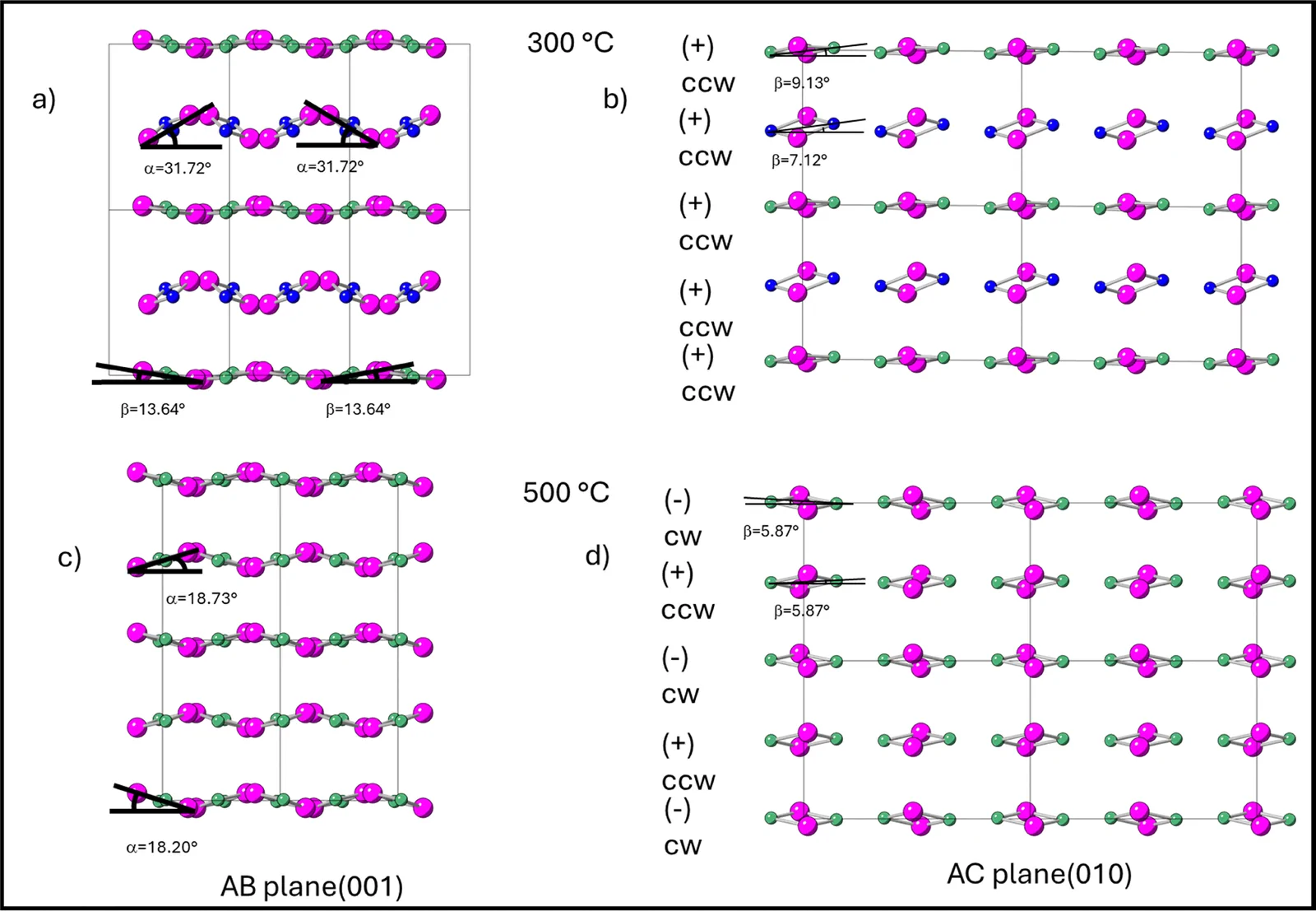
Projections along the AB and AC planes at 300 °C, (a) and (b), and 500 °C (c) and (d), respectively to highlight the Nb_2_O_10_ dimers. For (a) and (b) Nb(1) as olive spheres, Nb(2) as blue spheres and connecting oxygen atoms in magenta spheres. For (c) and (d) all Nb atoms as olive spheres. ccw and cw represent counter clockwise and clockwise canting, respectively.

The influence the polymorphic phase transition on the dielectric properties *via* DS is subtle and difficult to detect because of significant interference from space charge effects that dominate the DS data above ∼350 °C due to increasing (leakage) mixed conductivity. This is noticeable in both the permittivity and tan *δ* data where (above ∼400 °C) both decrease with increasing (fixed) frequency for a given temperature, Fig. 6. Analysing higher frequency DS data can be useful in reducing the level of space charge contribution and the 250 kHz DS data show an increase in permittivity from ∼28 at RT to a plateau value of ∼60 between 400 and 500 °C, Fig. 6(c). The subsequent upturn above 600 °C is due to the interference of space charge effects at these higher temperatures for this higher frequency (250 kHz) measurement. These higher (fixed) frequency DS measurements at 250 kHz show good reversibility on heating and cooling in the vicinity of phase transition at ∼420 °C, Fig. 6(c) and (d).

To avoid space charge effects and to probe the bulk properties of SrNb_2_O_6_, *M*″ spectroscopic plots from IS data were analysed. This shows an increase in bulk capacitance (and therefore permittivity) as the phase transition is approached and the existence of a broad maximum at ∼450 °C with a shallow decline at higher temperatures, Fig. 7(e). Thus, there is no ferroelectric to paraelectric phase transition in SrNb_2_O_6_. There is a modest increase in permittivity by a factor of two between m-SrNb_2_O_6_ at RT and o-SrNb_2_O_6_ at ∼600 °C; however, dielectric anomalies previously reported near RT and/or above 300 °C are more likely to be attributed to space charge effects associated with moisture up take near RT and leakage conductivity in the ceramics at >300 °C, respectively as discussed below.

The conductivity of SrNb_2_O_6_ ceramics show linear, Arrhenius type behaviour from 300 to 700 °C Fig. 7(c) with *E*_a_ ∼ 0.8 eV. There is evidence of mixed electronic (p-type) and oxide ion conduction (*t*_o_) from a combination of IS, Fig. 7(a), and EMF, SI Fig. S4, measurements. Given the anion lattice is an ∼hcp arrangement of oxide ions, the oxide ion conduction presumably occurs *via* a vacancy mechanism. The source and level of the oxygen deficiency is currently unknown and requires further investigation but is presumably associated with volatilisation of a low level of SrO during the sintering of the ceramics at 1350 °C and/or due to acceptor-type impurities in the reagents used in the solid-state reaction. The presence of a low level of oxide ion vacancies 
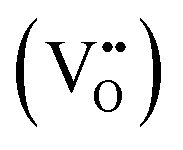
 in SrNb_2_O_6_ ceramics is also consistent with the sensitivity towards hydration at lower temperatures. It is well established that proton conduction can arise in oxides *via* the mechanism below,^32^ using the Kroger–Vink notation,
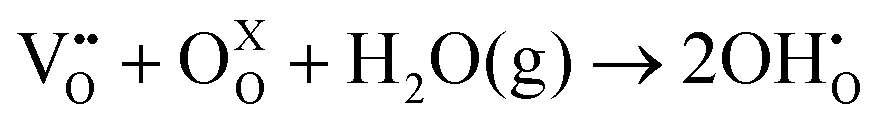
and further supports our observation of anomalously high tan *δ* near RT, Fig. 6(f).

This hydration phenomenon explains the origin of a peak in permittivity and tan *δ* near RT in previous studies^23,24^ where dehydration occurs close to RT but the temperature(s) associated with the dielectric anomalies is dependent on the level of oxide ion vacancies in the samples and on the humidity in the laboratory where the experiments were performed. In addition, the peak in permittivity observed at ∼300 °C in another study^25^ is attributed to space charge effects associated with the mixed ionic/electronic conduction where the level of oxide ion vacancies is dependent on the purity of the raw reagents used in the synthesis (*i.e.* the level of aliovalent impurities) and/or the sintering conditions (*i.e.* temperature and/or time) to limit volatilisation of SrO. This study reveals the important role that the level and hydration of oxide ion vacancies have in nominally stoichiometric ‘SrNb_2_O_6_’ ceramics to create extrinsic polarisation mechanisms that can give rise to anomalies in DS data that are mistaken to be signs of polymorphic phase transitions and/or ferroelectricity.^23–25^ It also highlights, the importance of considering the purity of the reagents used and the solid-state reaction and ceramic sintering conditions used when attempting to prepare a ‘stoichiometric’, dielectric compound such as SrNb_2_O_6_.

## Conclusions

VTXRD data for SrNb_2_O_6_ prepared by a solid-state route display the presence of a monoclinic (space group *P*2_1_/*c*) to orthorhombic (space group *Pmcn*) polymorphic phase transition between ∼400 to 500 °C with the temperature being established by DSC as ∼420 °C. The phase transition relieves overbonded Nb(2) cations and a combination of a reduction in the buckling and canting of Nb_2_O_10_ dimers drives a NTE of the *a*-axis as the m- to o-phase transition is approached. There is no ferroelectric behaviour in SrNb_2_O_6_. A combination of DS, IS and EMF measurements show it to be a modest dielectric with permittivity ∼28 and tan *δ* ∼ 0.003 at RT and a mixed p-type and oxide ion conductor at higher temperatures (>300 °C). The origin(s) of oxygen vacancies remains unknown and requires further investigation, but they are responsible for low levels of hydration (and therefore proton conduction) near RT that can give rise to space charge effects and produce anomalously high tan *δ* and/or peaks in permittivity near RT.

## Conflicts of interest

There are no conflicts to declare.

## Supplementary Material

TC-014-D6TC01467D-s001

## Data Availability

The data that support the findings of this study are available from the corresponding authors, Sadi Ege Parim or Derek Sinclair upon reasonable request. Supplemantry information (SI) is available. See DOI: https://doi.org/10.1039/d6tc01467d.
