# Supplementary material for: Polymorphism, negative thermal expansion and electrical properties of SrNb2O6
Source: J Mater Chem C Mater. 2026 Sep 16;14(41):20028–39. doi: 10.1039/d6tc01467d (PMC13637028; doi:10.1039/d6tc01467d)
Supplement: Supplementary file 1 [file TC-014-D6TC01467D-s001.pdf]

Supplementary Information (SI) for Journal of Materials Chemistry C.
This journal is © The Royal Society of Chemistry 2026

Supplementary Information

Figures

SI Figure 1: (a) to (f) VTXRD data for $SrNb_2O_6$ from 100 to 600 °C, respectively and wR as a function of temperature (g).

SI Figure 2: Back scattered electron images of $SrNb_2O_6$ ceramics sintered at 1275 and 1350 °C, (a) and (b) respectively. Elemental maps from EDX analysis for Sr, Nb and O for a ceramic sintered at 1350 °C, (c), (d) and (e), respectively.

SI Figure 3: RT XRD data for $SrNb_2O_6$ quenched in liquid nitrogen from 1000 °C (a) and compared to a slow cooled sample from the sintering temperature of 1350 °C (b).

SI Figure 4: Oxide ion transport number, $t_{ion}(O^{2-})$, versus temperature for $SrNb_2O_6$ ceramics sintered at 1350 °C.

Tables

SI Table 1: Rietveld refinement results of atomic coordinates, ion occupancies and thermal parameters for (a) m-$SrNb_2O_6$ at RT, and (b) o-$SrNb_2O_6$ at 700 °C.

SI Table 2: Rietveld refinement results for lattice parameters (a-c), tilt angle (β), unit cell volume (V), theoretical densities (ρ_{theo}), and weighted R value (w_R) from VTXRD data between RT and 700 °C

SI Table 3: Representative bond lengths and bond angles for m- and o-$SrNb_2O_6$ at (a) RT and (b) 700 °C.

SI Figures and Tables

Figures

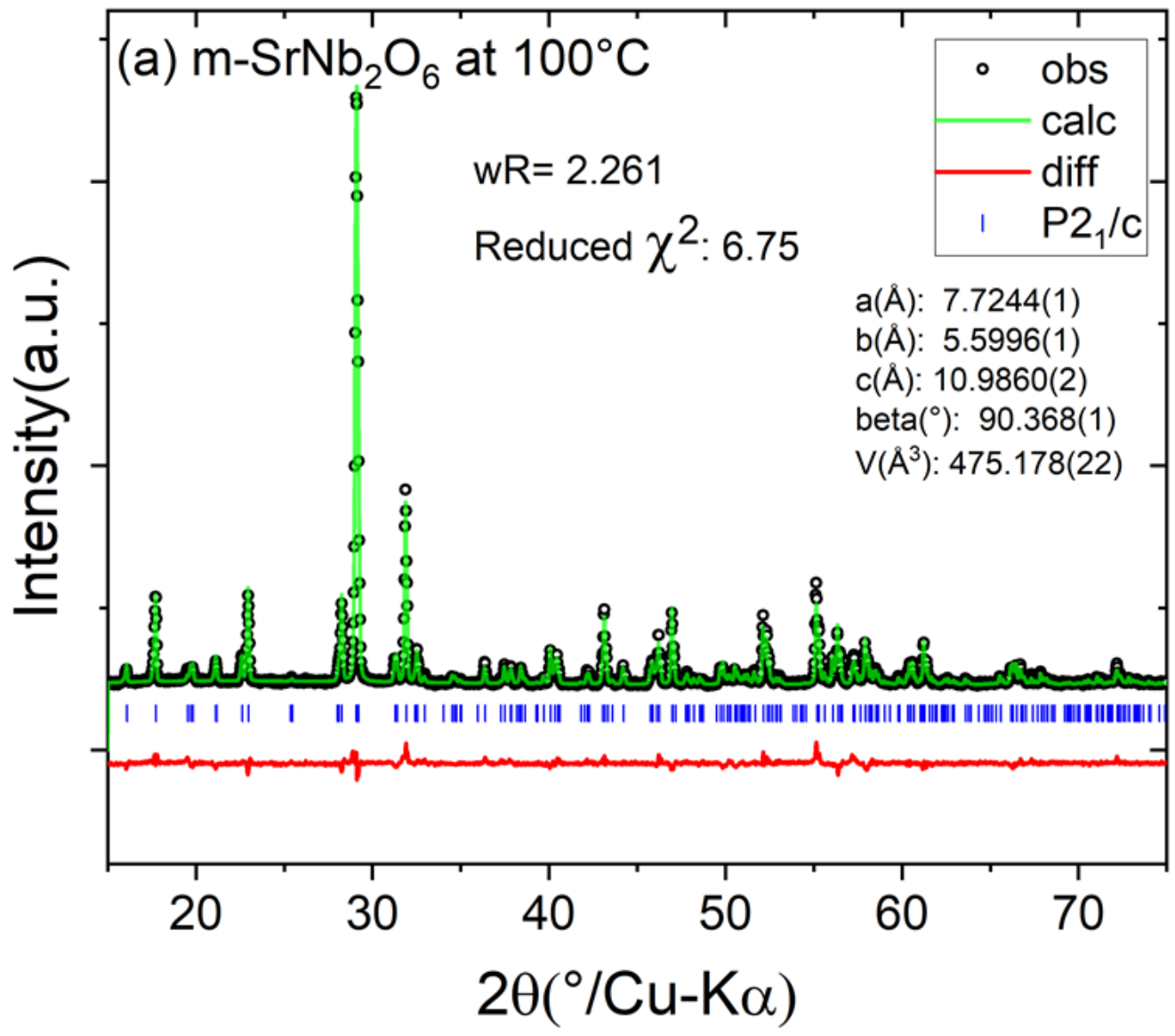

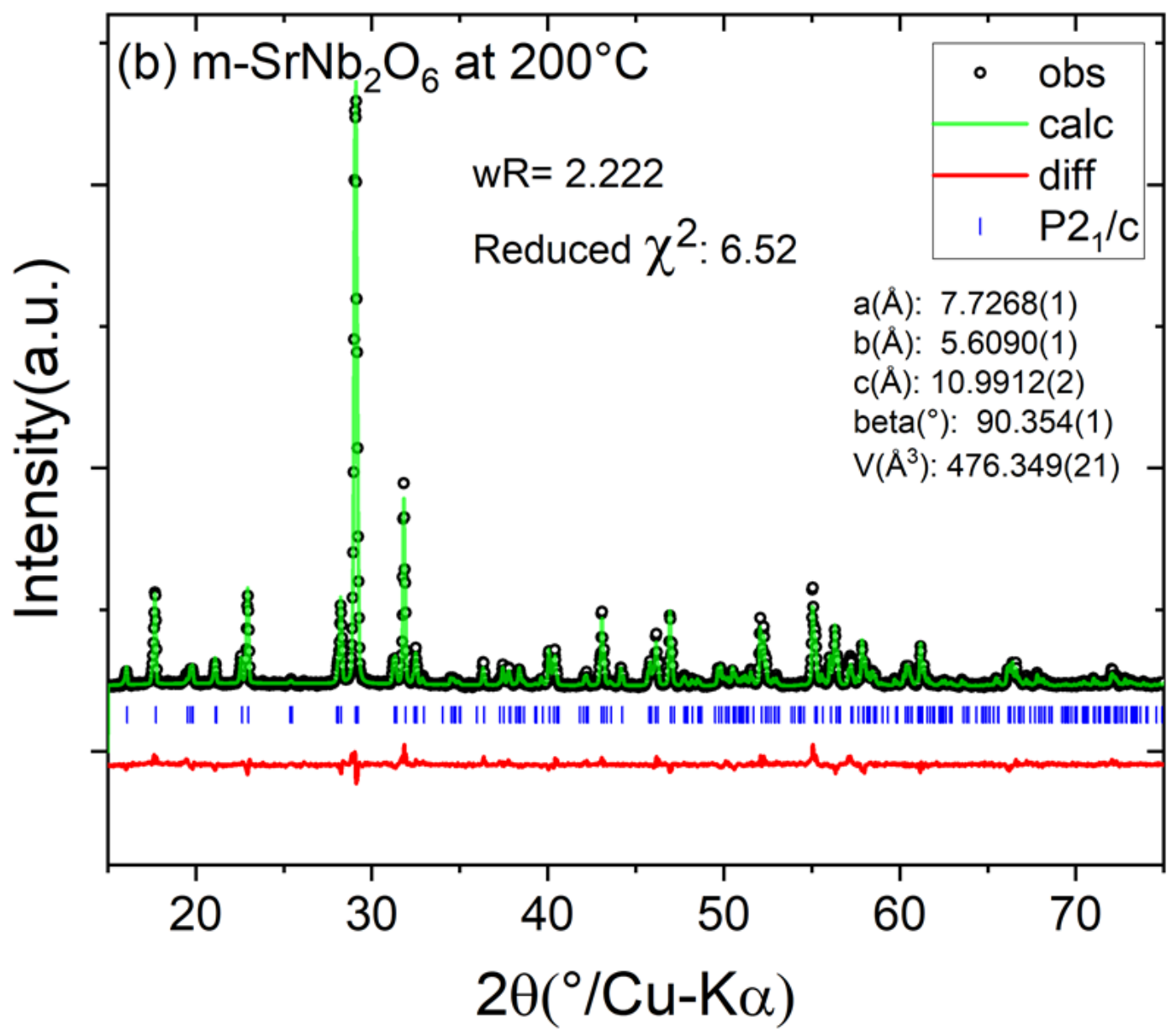

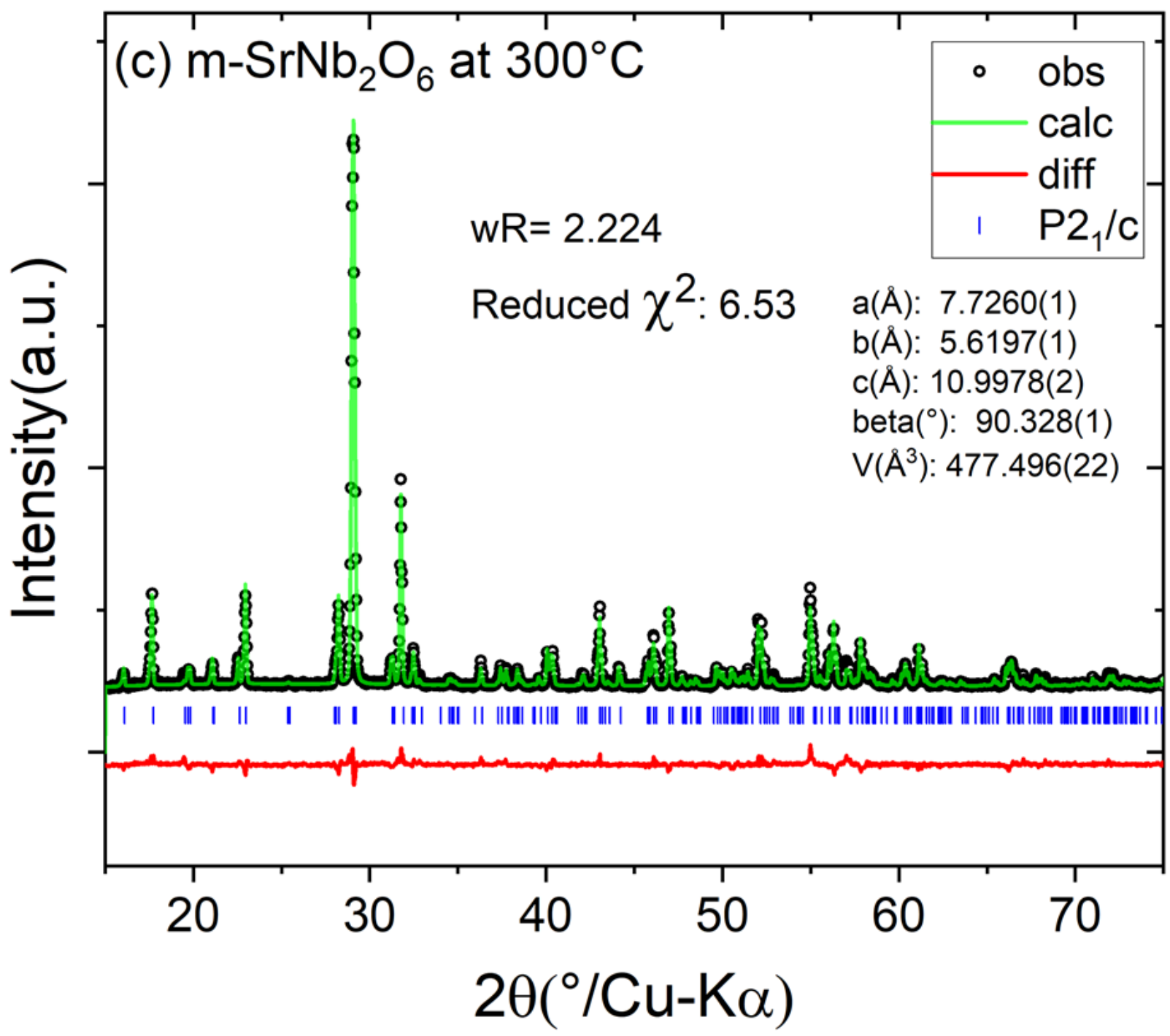
(c) m-SrNb2O6 at 300°C
obs
calc
diff
P21/c
wR= 2.224
Reduced χ2: 6.53
a(Å): 7.7260(1)
b(Å): 5.6197(1)
c(Å): 10.9978(2)
beta(°): 90.328(1)
V(Å3): 477.496(22)
Intensity(a.u.)
20
30
40
50
60
70
2θ(°/Cu-Kα)

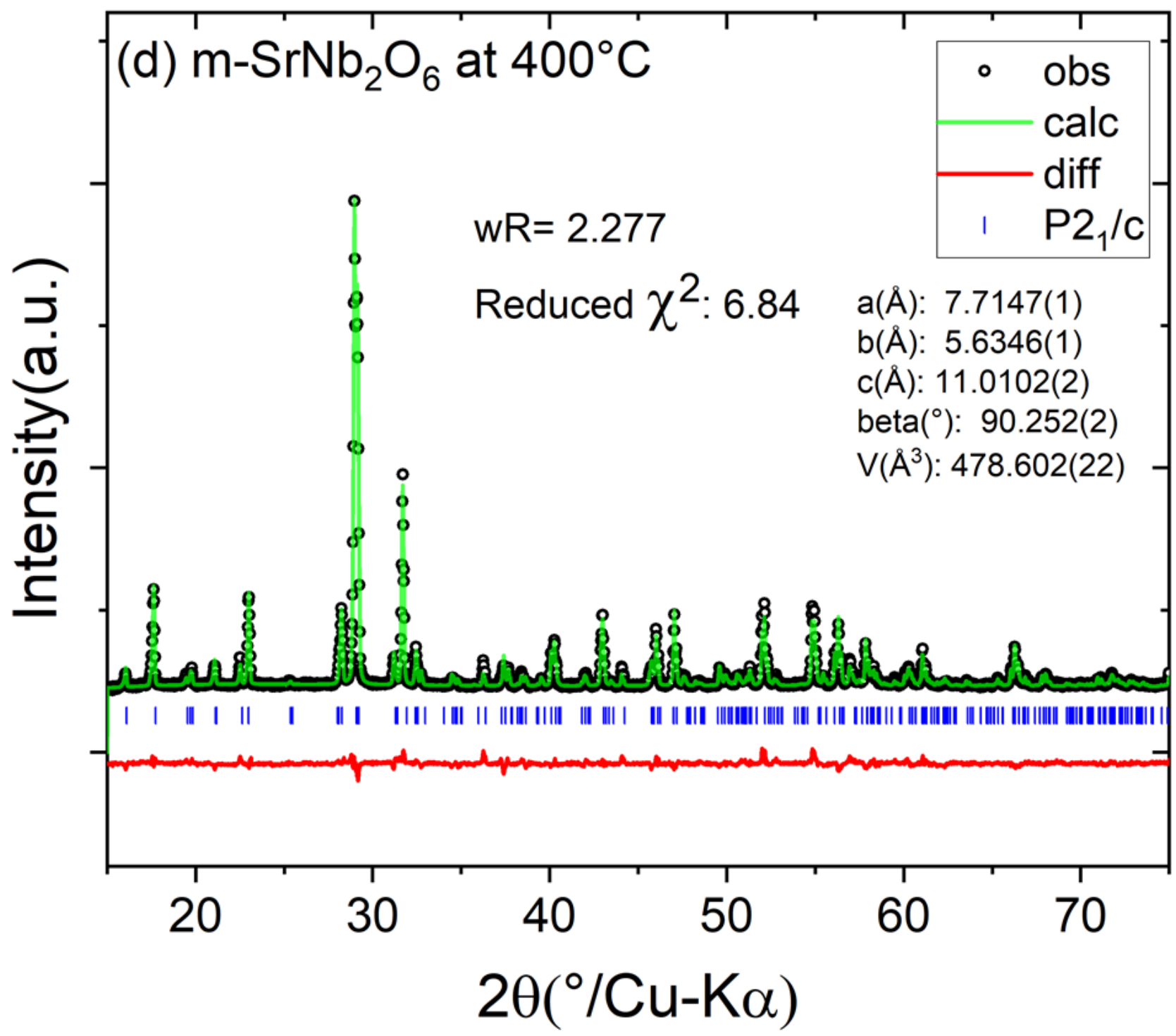
(d) m-SrNb2O6 at 400°C
obs
calc
diff
P21/c
wR= 2.277
Reduced χ2: 6.84
a(Å): 7.7147(1)
b(Å): 5.6346(1)
c(Å): 11.0102(2)
beta(°): 90.252(2)
V(Å3): 478.602(22)
Intensity(a.u.)
20
30
40
50
60
70
2θ(°/Cu-Kα)

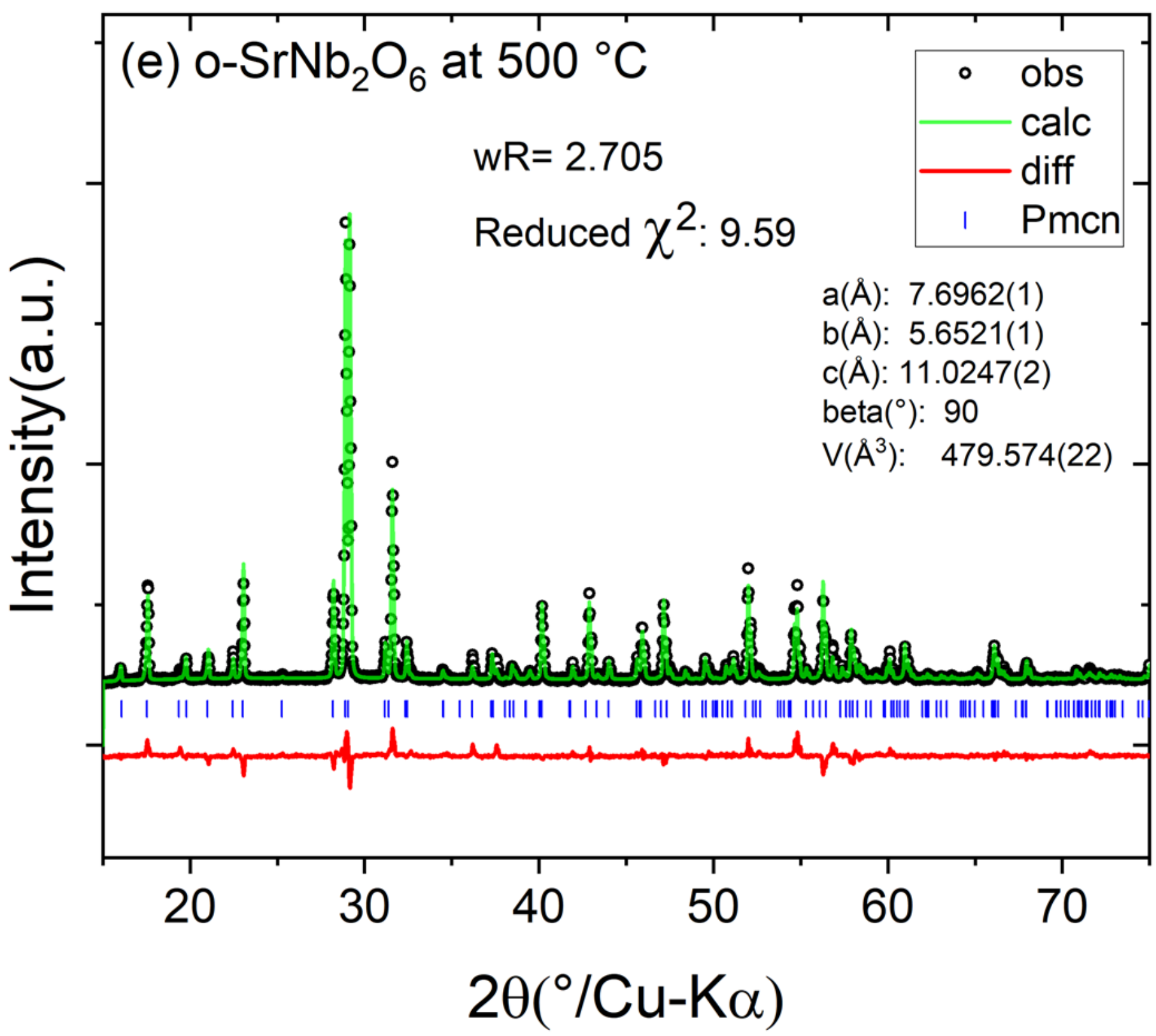

(e) o-$SrNb_2O_6$ at 500 °C
wR= 2.705
Reduced χ^2: 9.59
obs
calc
diff
Pmcn
a(Å): 7.6962(1)
b(Å): 5.6521(1)
c(Å): 11.0247(2)
beta(°): 90
V($Å^3$): 479.574(22)
Intensity(a.u.)
20
30
40
50
60
70
2θ(°/Cu-Kα)

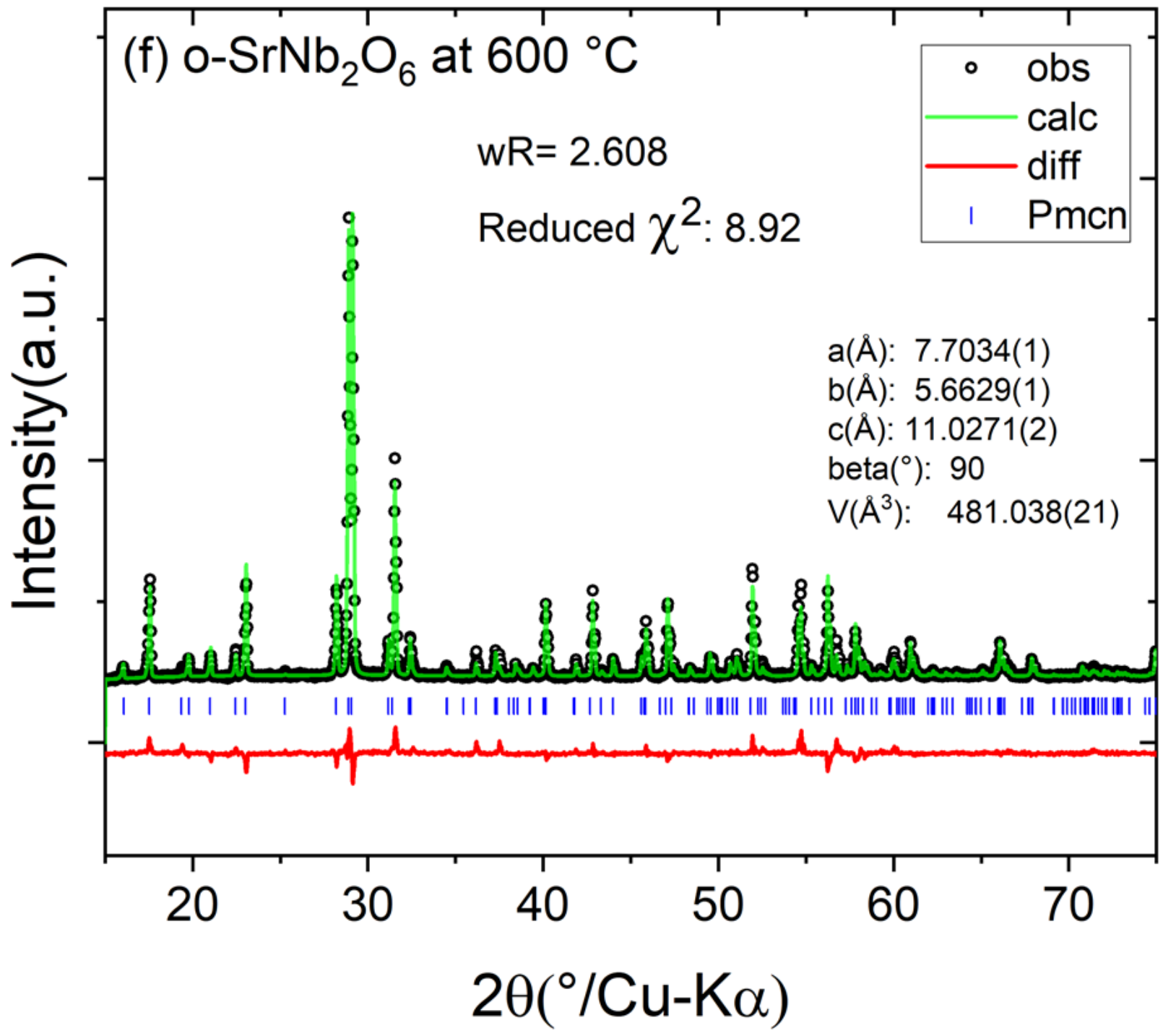

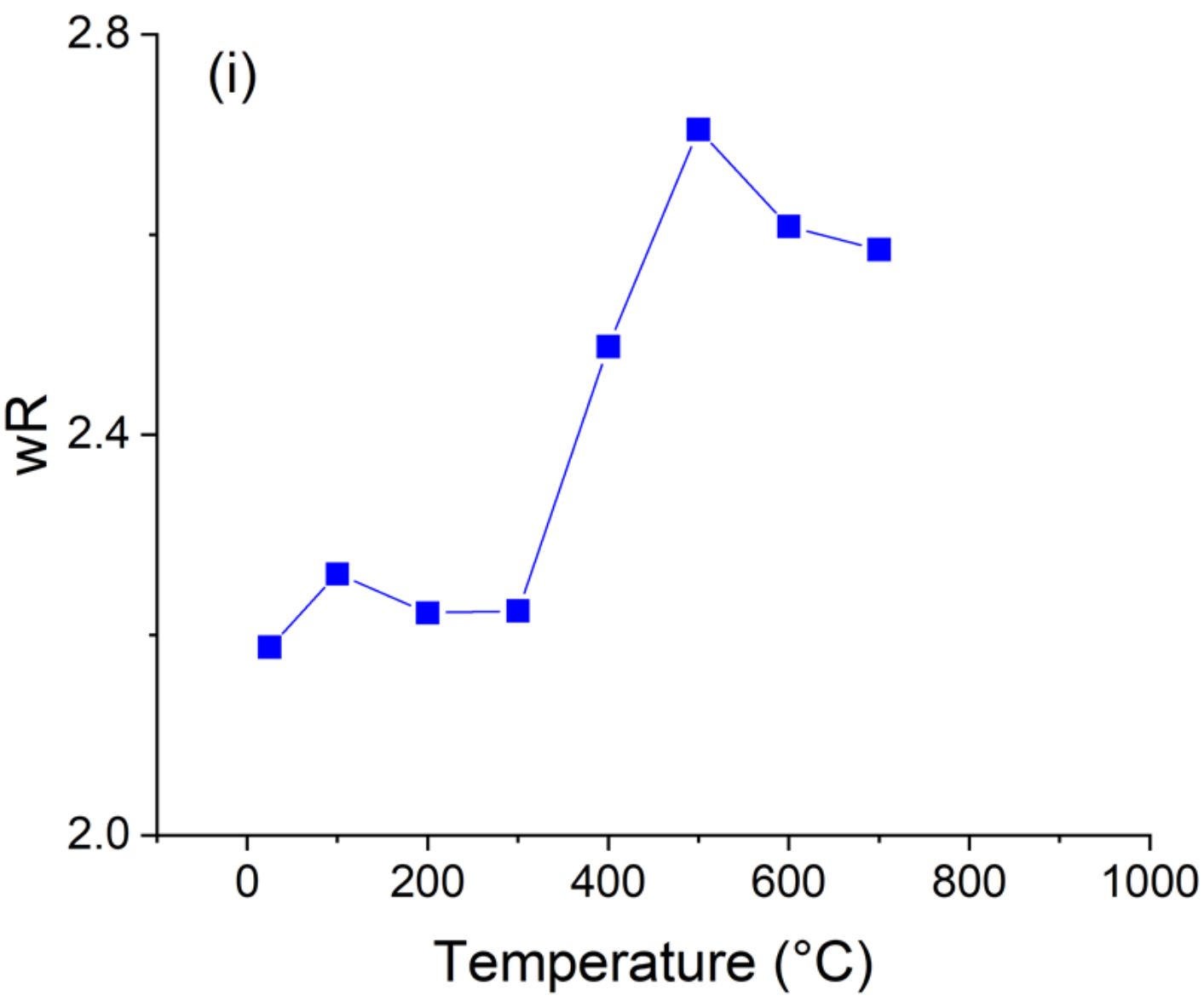

SI Figure 1: (a) to (f) VTXRD data for $SrNb_2O_6$ from 100 to 600 °C, respectively and wR as a function of temperature (g).

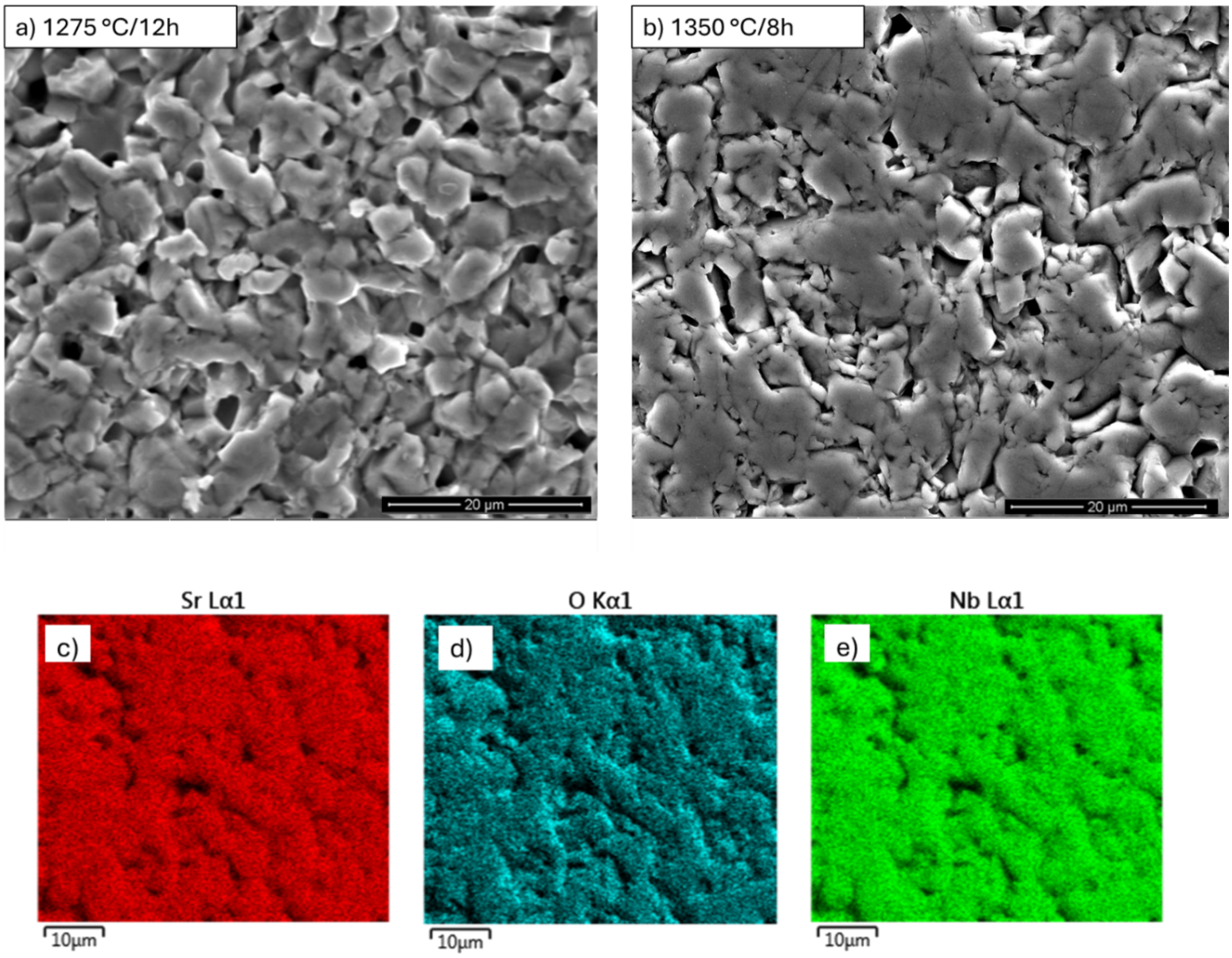

SI Figure 2: Back scattered electron images of $SrNb_2O_6$ ceramics sintered at 1275 and 1350 °C, (a) and (b) respectively. Elemental maps from EDX analysis for Sr, Nb and O for a ceramic sintered at 1350 °C, (c), (d) and (e), respectively.

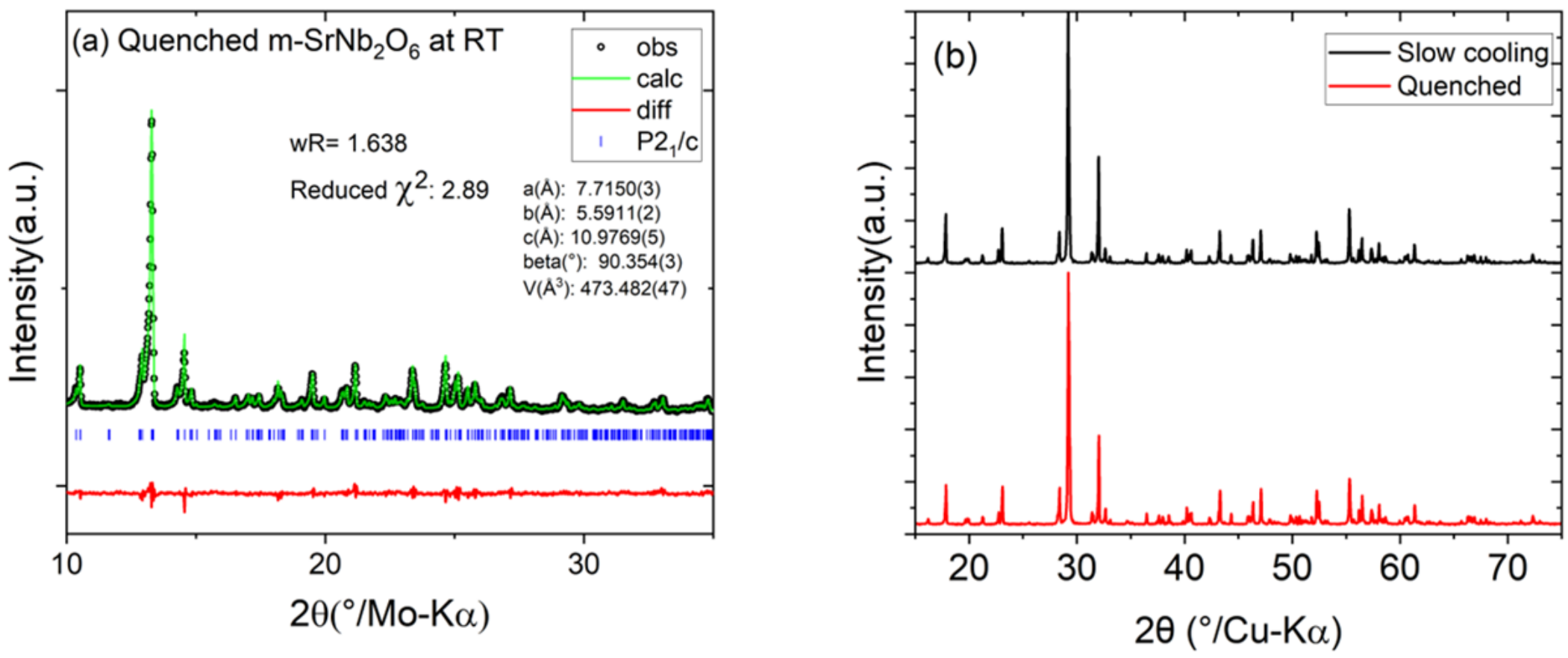

SI Figure 3: RT XRD data for $SrNb_2O_6$ quenched in liquid nitrogen from 1000 °C (a) and compared to a slow cooled sample from the sintering temperature of 1350 °C (b).

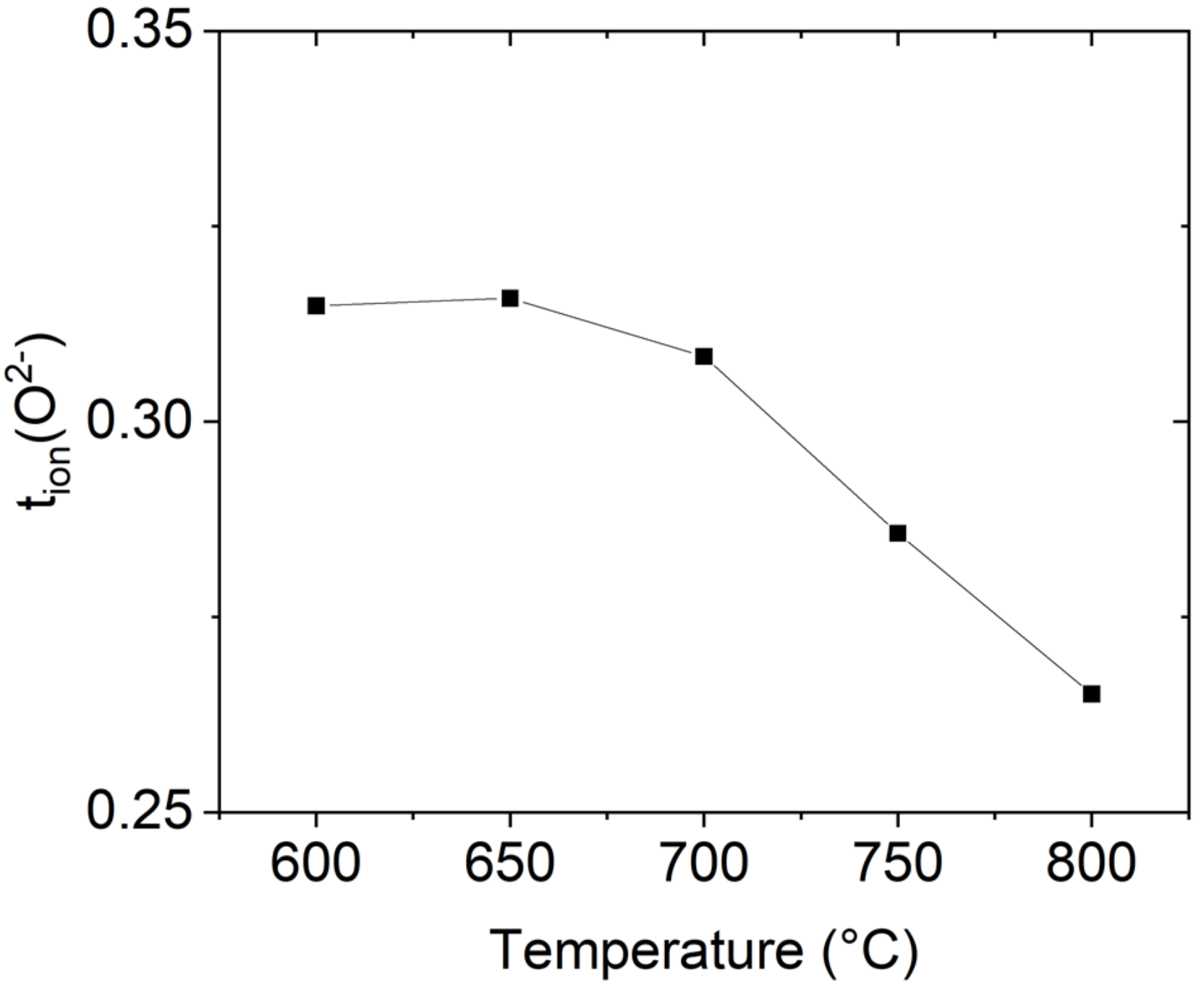

SI Figure 4: Oxide ion transport number, $t_{ion}(O^{2-})$, versus temperature for $SrNb_2O_6$ ceramics sintered at 1350 °C.

Tables

SI Table 1: Rietveld refinement results of atomic coordinates, ion occupancies and thermal parameters for (a) m-$SrNb_2O_6$ at RT, and (b) o-$SrNb_2O_6$ at 700 °C.

a)		x (Å)	y (Å)	z (Å)	Uiso (Å^2)	Occupancy
Sr1	Sr^{2+}	0.2540(5)	0.5310(8)	0.03952(23)	0.0149(14)	1
Nb1	Nb^{5+}	0.0169(4)	0.0245(14)	0.1432(3)	0.0156(15)	1
Nb2	Nb^{5+}	0.5269(4)	0.4642(14)	0.6415(3)	0.0130(15)	1
O1	O^{2-}	0.038(4)	0.205(4)	0.9812(26)	0.0218(29)	1
O2	O^{2-}	0.449(4)	0.309(4)	0.4823(25)	0.0218(29)	1
O3	O^{2-}	0.0478(28)	0.383(4)	0.2130(19)	0.0218(29)	1
O4	O^{2-}	0.4426(28)	0.114(5)	0.7112(21)	0.0218(29)	1
O5	O^{2-}	0.2673(29)	0.986(4)	0.1512(12)	0.0218(29)	1
O6	O^{2-}	0.754(4)	0.1156(29)	0.1279(15)	0.0218(29)	1
Bond Valence Sum (vu)						
	Sr-O		Nb1-O		Nb2-O	
	1.99		4.89		5.52	

b)	x (Å)	y (Å)	z (Å)	Uiso (Å^2)	Occupancy

Sr	Sr^{2+}	0.25	0.0256(14)	0.03408(29)	0.0474(17)	1
Nb	Nb^{5+}	0.0042(3)	0.0284(4)	0.35564(19)	0.0232(8)	1
O1	O^{2-}	0.5468(18)	0.2923(15)	0.0188(15)	0.0415(31)	1
O2	O^{2-}	0.5499(16)	0.3792(21)	0.2888(11)	0.0415(31)	1
O5	O^{2-}	0.25	0.510(5)	0.1524(14)	0.0415(31)	1
O6	O^{2-}	0.25	0.061(4)	0.3705(16)	0.0415(31)	1

SI Table 2: Rietveld refinement results for lattice parameters (a-c), tilt angle (β), unit cell volume (V), theoretical densities (ρ_{theo}), and weighted R value (w_R) from VTXRD data between RT and 700 °C

T (°C)	a (Å)	b (Å)	c (Å)	Beta (°)	Volume (Å^3)	d (g/cm^3)	wR(%)
RT	7.7216(1)	5.5934(1)	10.9829(2)	90.374(2)	474.348(22)	5.173	2.188
100	7.7244(1)	5.5996(1)	10.9860(2)	90.368(1)	475.178(22)	5.163	2.261
200	7.7268(1)	5.6090(1)	10.9912(2)	90.354(1)	476.349(21)	5.150	2.222
300	7.7260(1)	5.6197(1)	10.9978(2)	90.328(1)	477.496(22)	5.138	2.224
400	7.7142(1)	5.6344(1)	11.0144(2)	90.255(2)	478.558(23)	5.127	2.488
500	7.6962(1)	5.6521(1)	11.0247(2)	90	479.574(22)	5.117	2.705
600	7.7034(1)	5.6629(1)	11.0271(2)	90	481.038(21)	5.101	2.608
700	7.7118(1)	5.6743(1)	11.0293(2)	90	482.638(20)	5.084	2.585

SI Table 3: Representative bond lengths and bond angles for m- and o-$SrNb_2O_6$ at (a) RT, (b) 300, (c) 500 and (d) 700 °C.

a)

Bond type	Bond length (Å)
Sr1- O1	2.550(27)
Sr1- O’1	2.701(29)
Sr1- O2	2.509(26)
Sr1- O’2	2.780(29)
Sr1- O3	2.625(22)
Sr1- O4	2.510(23)
Sr1- O5	2.827(21)
Sr1- O6	2.700(17)
Nb1- O1	1.920(21)
Nb1- O’1	2.053(28)
Nb1- O3	1.839(22)
Nb1- O’3	2.159(23)
Nb1- O5	1.947(23)
Nb1- O6	2.099(30)
Nb2- O2	1.870(26)
Nb2- O’2	2.039(27)
Nb2- O4	1.836(30)
Nb2- O’4	2.203(28)
Nb2- O5	2.027(27)
Nb2- O6	1.817(30)

Ions			Angle (°)
O1	Sr1	O2	77.9(5)
O1	Sr1	O4	109.5(9)
O2	Sr1	O4	66.2(8)
O1	Nb1	O3	105.3(11)
O1	Nb1	O5	99.9(10)
O3	Nb1	O5	100.9(8)
O2	Nb2	O4	108.6(11)
O2	Nb2	O6	90.4(10)
O4	Nb2	O6	93.7(7)
Sr1	O1	Nb1	142.9(11)
Sr1	O2	Nb2	139.3(12)
Sr1	O4	Nb2	125.9(11)

b)

Bond type	Bond length (Å)
Sr1- O1	2.571(27)
Sr1- O’1	2.588(26)
Sr1- O2	2.510(25)
Sr1- O’2	2.972(23)
Sr1- O3	2.633(19)
Sr1- O4	2.517(21)
Sr1- O5	2.863(19)
Sr1- O6	2.735(18)
Nb1- O1	1.957(25)
Nb1- O’1	2.106(24)
Nb1- O3	1.863(22)
Nb1- O’3	2.144(24)
Nb1- O5	1.947(23)
Nb1- O6	2.033(28)
Nb2- O2	1.776(25)
Nb2- O’2	2.095(21)
Nb2- O4	1.841(24)
Nb2- O’4	2.211(26)
Nb2- O5	2.019(23)
Nb2- O6	1.984(27)

Ions			Angle (°)
O1	Sr1	O2	76.4(5)
O1	Sr1	O4	111.1(8)
O2	Sr1	O4	68.3(8)
O1	Nb1	O3	105.0(11)
O1	Nb1	O5	95.3(10)
O3	Nb1	O5	98.4(8)
O2	Nb2	O4	109.6(11)
O2	Nb2	O6	84.3(7)
O4	Nb2	O6	92.0(7)
Sr1	O1	Nb1	139.2(11)
Sr1	O2	Nb2	144.5(11)
Sr1	O4	Nb2	126.8(12)

c)

Bond type	Bond length (Å)
2x Sr1- O1	2.475(14)
2x Sr1- O’1	2.706(14)
2x Sr1- O2	2.618(12)
Sr1- O5	3.001(27)
Sr1- O6	2.847(20)

Ions			Angle (°)
Nb1	O1	Sr4	140.9(7)
O1	Nb1	O2	105.4(5)
O1	Nb1	O5	98.4(7)
O2	Nb1	O5	98.1(7)
O1	Nb1	O6	83.8(6)
O2	Nb1	O6	87.6(5)

d)

Bond type	Bond length (Å)
2x Sr1- O1	2.460(17)
2x Sr1- O'1	2.749(13)
2x Sr1- O2	2.624(13)
Sr1- O5	3.042(28)
Sr1- O6	2.959(22)
Nb- O1	1.955(13)
Nb- O'1	2.104(15)
Nb- O2	1.838(12)
Nb- O'2	2.163(12)
Nb- O5	1.965(3)
Nb- O6	1.912(4)

Ions			Angle (°)
Nb1	O1	Sr4	141.4(7)
O1	Nb1	O2	105.5(5)
O1	Nb1	O5	99.4(8)
O2	Nb1	O5	97.3(7)
O1	Nb1	O6	80.8(6)
O2	Nb1	O6	86.0(6)
O5	Nb1	O6	176.5(9)
O1	Sr4	O1	79.2(5)
Nb1	O5	Nb1	172.1(15)
Nb1	O6	Nb1	165.0(12)
